# Oncogenic Role of SAMD4B in Breast Cancer Progression by Activating Wnt/β-Catenin Pathway

**DOI:** 10.3390/biom15101423

**Published:** 2025-10-07

**Authors:** Jia-Hui Li, Xin-Ya Wang, Huan-Xi Song, Xiao-Fei Nie, Li-Na Zhang

**Affiliations:** Beijing International Science and Technology Cooperation Base of Antivirus Drug, College of Chemistry and Life Science, Beijing University of Technology, Beijing 100124, China; ljhuiii@emails.bjut.edu.cn (J.-H.L.); wangxinya@emails.bjut.edu.cn (X.-Y.W.); songhuanxi@emails.bjut.edu.cn (H.-X.S.); niexiaofei@emails.bjut.edu.cn (X.-F.N.)

**Keywords:** SAMD4B, RNA-binding protein, Wnt/β-catenin pathway, mRNA stability, breast cancer

## Abstract

The Sterile alpha motif domain-containing protein 4 (SAMD4) family consists of two evolutionarily conserved and highly homologous RNA-binding proteins, SAMD4A and SAMD4B. Previous studies have established SAMD4A as a tumor suppressor that is downregulated in breast cancer, while the function of SAMD4B in tumorigenesis remains poorly defined. In this study, we observed that SAMD4B expression is upregulated in breast cancer. Functional assays demonstrated that SAMD4B facilitated breast cancer cell proliferation, migration, and invasion by inducing epithelial–mesenchymal transition (EMT). Furthermore, SAMD4B accelerated G1-to-S phase cell cycle progression by modulating p53 expression, collectively supporting an oncogenic function of SAMD4B in breast cancer. Mechanistically, we found that SAMD4B enhanced TCF/LEF transcriptional activity and upregulated the expression of β-catenin, Cyclin D1, c-Myc, and Axin2. Further investigations confirmed that SAMD4B activated the Wnt/β-catenin pathway by stabilizing *β-catenin* mRNA and increasing β-catenin protein expression level. Importantly, treatment with XAV-939, a specific Wnt/β-catenin pathway inhibitor, abrogated the pro-oncogenic effects of SAMD4B overexpression, including Wnt/β-catenin pathway activation, enhanced proliferation, and increased metastatic capacity. These results confirm that SAMD4B promotes the malignant phenotypes of breast cancer cells in a manner dependent on the Wnt/β-catenin pathway. In summary, our findings clarify that SAMD4B exerts an oncogenic role in breast cancer progression by activating the Wnt/β-catenin pathway. These data identify SAMD4B as a potential therapeutic target in breast cancer, although further in vivo investigations are required to validate its clinical relevance.

## 1. Introduction

As a highly prevalent cancer and the leading cause of cancer-related death in women globally [1], breast cancer presents a growing challenge in recent years. Despite the widespread use of surgery and chemotherapy, the identification of novel diagnostic markers and therapeutic targets remains a critical unmet need in molecular-targeted therapy. Therefore, understanding the precise molecular mechanisms that drive breast cancer progression is of crucial importance [2,3].

Post-transcriptional regulation is a key and multi-layered step in eukaryotic gene expression, encompassing processes such as the splicing and processing of hnRNA, nuclear export of mature mRNA, and the control of mRNA stability and degradation [4]. RNA-binding proteins (RBPs) are central to this regulation, orchestrating every step of an mRNA’s life cycle [5]. Dysregulation of RBPs is closely associated with various human diseases, such as neurodegenerative diseases [6,7]. Notably, accumulating evidence confirms that aberrant RBP expression in cancers, particularly breast cancer, dysregulates the stability and translation of target mRNAs, thereby modulating the expression of key oncogenes and tumor suppressors and influencing tumor initiation and progression [8,9,10,11,12].

The sterile alpha motif domain-containing protein 4 (SAMD4) family is a new class of highly specific, evolutionarily conserved RBPs that mediate post-transcriptional regulation and translational inhibition in eukaryotes [13,14,15]. This family includes two homologous mammalian members, SAMD4A (Smaug1) and SAMD4B (Smaug2), both of which possess a conserved RNA-binding SAM domain that binds to stem-loop structures located in the 3′-UTR of target mRNAs to mi [16]. In *Drosophila*, Smaug (homolog of SAMD4) recruits CCR4 and POP2 deadenylases to target mRNAs, triggering their deadenylation and degradation [17]. Smaug also post-transcriptionally regulates maternal mRNAs such as *Hsp83* and *Nanos* by directly binding to them or recruiting other proteins, thereby controlling their stability and translation [18,19,20,21]. Similarly, mammalian SAMD4 members are involved in diverse critical biological processes. For instance, SAMD4 family members can inhibit human hepatitis B virus (HBV) replication through binding viral RNA and inducing its decay [22]. SAMD4A can regulate skeleton development by translationally inhibiting Mig6 [23]. Recent studies further indicate that SAMD4A regulates cardiomyocyte lineage commitment from human embryonic stem cells (hESCs) by post-transcriptionally regulating FGF2 [24], and also suppresses abdominal aortic aneurysm development and vascular smooth muscle cell (VSMC) phenotypic transformation by targeting KDM2B [25].

Interestingly, emerging evidence has linked dysregulated expression of SAMD4 family members to cancer progression. For instance, SAMD4A is downregulated in breast cancer, and its upregulation inhibits breast cancer progression and angiogenesis [26]. Conversely, SAMD4A is elevated in gastric cancer and associated with poor prognosis, suggesting an oncogenic role in this context [27]. However, in contrast to SAMD4A, the direct role of SAMD4B in tumorigenesis remains unelucidated. Previous studies indicate that SAMD4B functions as a potential transcriptional regulator, inhibiting the activities of AP-1, p53, and p21 to influence cell cycle and apoptosis [28]. Its expression is also post-transcriptionally suppressed by miR-451 in colorectal cancer, which inhibits cell proliferation and promotes apoptosis [29]. Recent research demonstrates that under sensing arginine deficiency, SAMD4B is released by Bcl2-associated athanogene 2 (BAG2) and promotes β-catenin degradation to stabilize ATF4, thereby promoting tumor cell survival [30]. Despite these findings establishing an indirect involvement of SAMD4B in tumorigenesis, its specific function and the molecular regulatory mechanisms in breast cancer pathogenesis remain unelucidated.

The epithelial–mesenchymal transition (EMT) is a well-established driver of tumor initiation, invasion, and metastasis. During this process, epithelial cells acquire enhanced migration, invasion, and drug resistance [31]. EMT is regulated by multiple signaling pathways, including TGF-β, PI3K/AKT, MAPK/ERK, NF-κB, and notably the Wnt/β-catenin pathway [32]. The Wnt/β-catenin pathway plays a particularly critical role in cancer development and metastasis, serving as a key mediator of EMT [33]. Its aberrant activation in various cancers promotes tumorigenesis and metastasis through this mechanism [34]. Despite its significance, the regulatory relationship between SAMD4B and either EMT or the Wnt/β-catenin pathway has not been reported in breast cancer.

In our present work, we comprehensively investigated the functional role and molecular regulatory mechanisms of SAMD4B in the process of breast cancer initiation and progression. Our findings demonstrated that SAMD4B promotes breast cancer cell proliferation, metastasis, and the G1-S phase transition of the breast cancer cell cycle through the activation of the Wnt/β-catenin signaling pathway. These results preliminarily provide in vitro evidence that SAMD4B functions as an oncogene to drive breast cancer progression, and further determined that it may serve as a potential biomarker and promising therapeutic target for breast cancer. Nevertheless, additional in vivo studies remain necessary to verify its oncogenic function and the regulatory mechanisms through which it exerts effects in breast cancer.

## 2. Materials and Methods

### 2.1. Cell Lines and Cell Culture

Human normal breast epithelial cell line MCF-10A (Resource No. 3101-HUMGNHu50) was purchased from the Cell Bank of the Typical Culture Preservation Committee of the Chinese Academy of Sciences (Shanghai, China). Human breast cancer cell lines MCF-7 (Resource No. 1101HUM-PUMC000013) and MDA-MB-231 (Resource No. 1101HUM-PUMC000014) were obtained from the Cell Resource Center, Institute of Basic Medical Sciences, Chinese Academy of Medical Sciences (Beijing, China). MCF-7 and MDA-MB-231 cells were grown in high-glucose Dulbecco’s Modified Eagle Medium (DMEM; Cat. No. SH30022.01B, Hyclone, Logan, UT, USA) supplemented with 10% fetal bovine serum (FBS; Cat.No. 10270-106, Gibco, Grand Island, NY, USA) and 1% penicillin-streptomycin (Cat. No. SV30010, Hyclone). For MCF-10A cells, the culture medium was DMEM/F12 (Cat. No. SH30023.01, Hyclone), also containing 10% FBS and 1% penicillin-streptomycin. All three cell lines were incubated at 37 °C in a humidified environment containing 5% CO_2_. Upon reaching approximately 80–90% confluency, cells were harvested via digestion with 0.25% trypsin-EDTA (Cat. No. SH30042.01, Hyclone) and prepared for subsequent experiments.

### 2.2. Construction of Stable Breast Cancer Cell Lines

Specific short hairpin RNA (shRNA) sequences targeting *SAMD4B* were cloned into the pLVX-shRNA2-Puro lentiviral vector (Cat. No. 632177, Takara Bio, San Jose, CA, USA), whereas the full-length SAMD4B cDNA was inserted into the pCDH-CMV-MCS-EF1-Puro lentiviral vector (Cat. No. CD812A-1, System Biosciences, Palo Alto, CA, USA). These lentiviral constructs (for *SAMD4B* knockdown or overexpression) and their corresponding control vectors were transfected into HEK-293T cells to generate lentiviral particles. MCF-7 and MDA-MB-231 cells were then infected with four lentiviral groups: SAMD4B knockdown (shSAMD4B), negative control (shNC), SAMD4B overexpression (SAMD4B), and empty control (Vector). At 24~48 h post-infection, cells were subjected to selection with 3.5 μg/mL puromycin (Cat. No. P8230, Solarbio, Beijing, China) in DMEM medium for approximately two weeks. The efficiency of *SAMD4B* knockdown and overexpression was subsequently validated at the mRNA level using RT-qPCR and at the protein level via Western Blotting, respectively.

### 2.3. Reverse Transcription (RT)-qPCR

Total RNA was isolated from cells with an RNA purification kit (Cat. No. DP430, TIANGEN, Beijing, China). Complementary DNA (cDNA) was synthesized utilizing the PrimerScript RT Reagent Kit (Cat. No. RR047A, Takara, Dalian, China) following the manufacturer’s protocols. qPCR analyses were conducted in triplicate for each target gene using SYBR Green Mix Kit (Cat. No. A301, GenStar, Beijing, China) on a Stratagene Mx3000P qPCR system (Agilent, Palo Alto, CA, USA). *GAPDH* mRNA served as an internal standard. The thermal cycling parameters were as follows: initial denaturation at 95 °C for 2 min; followed by 40 cycles of denaturation at 95 °C for 15 s, annealing at 60 °C for 30 s, and extension 72 °C for 30 s; with a final dissociation stage consisting of 95 °C for 1 min, 60 °C for 30 s, and 72 °C for 30 s. Relative gene expression levels were calculated using the 2^−△△Ct^ method and normalized against the control group. Detailed sequences of the primers used for qPCR are provided in Table 1.

### 2.4. Western Blotting

Protein extraction was performed using RIPA lysis buffer (Cat. No. WB-0072, Genview, Beijing, China), and protein concentration was determined with a BCA Protein Assay Kit (Cat. No. BCA01, Genview). After protein denaturation, equal amounts of samples (40 μg) were separated by 10~12% SDS-PAGE and then transferred onto PVDF membranes (Cat. No. IPVH00010, Millipore, Billerica, MA, USA). After blocking with 5% non-fat milk (Cat. No. PS112, Epizyme, Shanghai, China) for 1 h at room temperature, the membranes were incubated overnight at 4 °C with the following primary antibodies: SAMD4B (1:1000, 17723-1-AP, Proteintech, Wuhan, China), p53 (1:1000, 9282T, CST, Danvers, MA, USA), p21 (1:1000, 2947T, CST), Cyclin D1 (1:1000, WL01435a, Wanleibio, Shenyang, China), CDK4 (1:1000, WL02274, Wanleibio), Cyclin E (1:1000, WL01072, Wanleibio), CDK2 (1:1000, WL01543, Wanleibio), E-cadherin (1:1000, 20874-1-AP, Proteintech), N-cadherin (1:1000, 22018-1-AP, Proteintech), Vimentin (1:1000, 17723-1-AP, Proteintech), Snail (1:1000, WL01863, Wanleibio), Slug (1:1000, 12129-1-AP, Proteintech), MMP-2 (1:1000, 10373-2-AP, Proteintech), MMP-9 (1:1000, 10375-2-AP, Proteintech), β-catenin (1:1000, 9562S, CST), Axin2 (1:500, 2151T, CST), c-Myc (1:1000, 5605T, CST), and β-actin (1:5000, TA-09, ZSGB-Bio, Beijing, China). The membranes were then incubated with horseradish peroxidase (HRP)-conjugated secondary antibodies (1:5000, ABGENT, San Diego, CA, USA) for 1 h at room temperature. Protein bands were visualized with an ECL chemiluminescent HRP substrate (Cat. No. WBKLS0100, Millipore) and captured using an automated chemiluminescence imaging system (Tanon 4200, Shanghai, China). β-actin served as the loading control. Band intensities were quantified using ImageJ 1.4k software, and the relative expression levels of each target protein were normalized to the control group.

### 2.5. MTT Assay

Cells were seeded in 96-well plates at a density of 2 × 10^3^ cells per well in triplicate. Cell proliferation was evaluated at 24, 48, 72, and 96 h post-seeding with an MTT Cell Proliferation Assay Kit (Cat. No. M1020, Solarbio). Briefly, at each time point, the culture medium was aspirated and replaced with 90 μL of fresh medium. 10 μL of MTT solution was added to each well, and the plates were incubated for an additional 4 h. Following careful removal of the supernatant, 110 μL of Formazan Solubilization Solution was added to each well, and the plates were gently shaken at a low speed for 10 min. The absorbance was determined at 490 nm using a microplate reader (BioTek, Winooski, VT, USA) to quantify the cell proliferation rate.

### 2.6. Plate Colony Formation Assay

Cells were seeded in 6-well plates at a density of 2 × 10^3^ cells per well in triplicate and cultured for approximately 15 days to allow colony formation. The resulting colonies were then fixed with 4% paraformaldehyde (800 μL/well, Cat. No. AR-0211, Genview) for 10 min, and then stained with 1% crystal violet solution (800 μL/well; Cat. No. 0528, Amresco, Solon, OH, USA) for 15 min. The plates were washed, air-dried, and the visible colonies were imaged and quantified.

### 2.7. Cell Cycle Analysis

Cells were seeded in 60 mm dishes, with each experimental condition set up in triplicate. When the cells reached 70–80% confluence, they were harvested and fixed overnight at 4 °C using 70% ice-cold ethanol. Following fixation, the cells were rinsed with PBS and then incubated at 37 °C for 30 min with 100 μL of PBS containing 100 μg/mL RNase A (Cat. No. R1030, Solarbio). After the RNase A treatment, the cells were stained in the dark: 300 μL of PBS supplemented with 50 μg/mL propidium iodide (PI; Cat. No. C0080, Solarbio) was added, and the mixture was incubated at room temperature for 30 min. The stained cell suspension was passed through a 300-mesh nylon screen to remove clumps, after which it was collected into flow cytometry tubes. Flow cytometric analysis was performed using a FACSCalibur flow cytometer (BD Biosciences, San Jose, CA, USA). Finally, the cell proportions in the G0/G1, S, and G2/M phases were determined by the ModFit LT 3.0 software (Verity Software House, Topsham, ME, USA).

### 2.8. Dual-Luciferase Reporter Gene Assay

Promoter sequences of p53 and p21 were retrieved from databases, such as the UCSC Genome Browser, by defining a 2 kb region upstream of their respective transcription start sites (TSSs) and extracting their corresponding sequences. These retrieved promoter sequences were then amplified via PCR using specific primers and genomic DNA as the template, followed by cloning into the pGL3-Basic reporter vector (Cat. No. E1751, Promega, Madison, WI, USA). Topflash and Fopflash luciferase reporter vectors were obtained from ZeyeBio (Cat. No. ZY1592, ZY1593, Shanghai, China). For luciferase assays, cells were seeded in 24-well plates and co-transfected with either p53/p21 promoter reporter constructs or Topflash/Fopflash vectors, together with the pRL-TK vector (serving as an internal control), using Chemifect transfection reagent (Cat. No. FR-01, FENGRBIO, Beijing, China). At 48 h post-transfection, the medium was discarded, and the cells were lysed with 120 μL of Cell Lysis Buffer for 20 min. Luciferase activities of p53/p21 promoter and Topflash/Fopflash were measured using the Dual-Luciferase Reporter Gene Assay Kit (Cat. No. FRT-02, FENGRBIO) according to the manufacturer’s instructions. Firefly luciferase activity for each reporter (p53/p21 promoter or Topflash/Fopflash) was normalized to the corresponding Renilla luciferase activity. Relative luciferase activity fold change refers to the ratio obtained by normalizing the luciferase activity triggered by SAMD4B against the luciferase activity triggered by the control.

### 2.9. Wound Healing Assay

Stable cells were seeded into 24-well plates at a density of 1 × 10^5^ cells per well, with each condition tested in triplicate. When the cell monolayer achieved 80% confluence, linear scratches were created by scraping across the cell layer using a sterile 10 μL pipette tip. After scratching, the culture medium was carefully aspirated and replaced with fresh DMEM. Images of the scratch areas were acquired at 0, 24, and 48 h post-scratching using an inverted light microscope (Olympus IX71, Tokyo, Japan). To evaluate cell migration capacity, the width of each scratch was measured at 24 h and 48 h using ImageJ software. Migration rates were determined by calculating the relative decrease in scratch width compared to the initial width measured at 0 h.

### 2.10. Transwell Assay

Transwell migration and invasion assays were conducted using 24-well Transwell chambers with an 8 μm pore size (Cat. No. PTEP24H48, Millipore, Billerica, MA, USA). For the migration assay, 4 × 10^4^ MCF-7 or 3 × 10^4^ MDA-MB-231 cells suspended in 200 μL serum-free medium were seeded into the upper chamber. For the invasion assay, 8 × 10^4^ MCF-7 or 6 × 10^4^ MDA-MB-231 cells in 200 μL serum-free medium were plated into the upper chamber, which had been precoated with 80 μL Matrigel (Cat. No. 356234, BD Biosciences, San Jose, CA, USA). The lower chamber was filled with 500 μL complete medium containing 20% FBS as a chemoattractant. After 24 h or 48 h of cultivation, non-migrated/non-invaded cells on the upper surface were carefully removed with a cotton swab. Cells that had migrated to or invaded the lower membrane were fixed with 4% paraformaldehyde for 30 min and subsequently stained with 1% crystal violet for an additional 30 min. Following washes with PBS, migrated and invaded cells were imaged under an optical microscope at 200× magnification and quantified by counting cells in randomly selected fields.

### 2.11. Immunofluorescence Analysis

Cells were seeded on glass coverslips that had been pre-placed in a 24-well plate. Upon reaching 70–80% confluence, the cells were transfected with either the pEGFP-SAMD4B recombinant plasmid or the pEGFP-C3 empty vector control. After 24 h of transfection, the cells were fixed with 4% paraformaldehyde (800 μL/well) for 30 min, followed by nuclear staining with DAPI solution (500 μL/well; Cat. No. C0060, Solarbio) for 10 min in the dark. After three washes with PBS, the coverslips were then mounted onto glass slides. Successfully transfected cells were visualized using a Nikon AX R confocal microscope (Tokyo, Japan).

### 2.12. mRNA Stability Analysis

SAMD4B-overexpressing cells and control cells were grown in a 12-well plate and treated with 5 μg/mL Actinomycin D (Cat. No. A4262, Sigma-Aldrich, Saint Louis, MO, USA) to block transcription. Cells were harvested at 0, 2, 4, and 6 h post-treatment. Total RNA was isolated using an RNA purification kit, and the abundance of *β-catenin* mRNA was quantified by RT-qPCR. The relative *β-catenin* mRNA level at 2, 4, and 6 h was calculated by normalization to the level at 0 h.

### 2.13. RNA Immunoprecipitation (RIP) Assay

Breast cancer cells were transiently transfected with Flag-tagged SAMD4B for 48 h and subsequently lysed using IP lysis buffer (Cat. No. P2179S, Beyotime, Shanghai, China). For immunoprecipitation, the cell lysates were incubated overnight at 4 °C with an anti-Flag antibody (Cat. No. 20543-1-AP, Proteintech) or a control IgG antibody (Cat. No. SA00001-2, Proteintech) that had been pre-conjugated to protein A/G magnetic beads (Cat. No. HY-K0202, MedChemExpress, NJ, USA). RNA associated with the immunoprecipitated SAMD4B complex was extracted using an RNA purification kit and reverse-transcribed into cDNA. Both semi-quantitative and quantitative RT-PCR analyses were then performed to detect *β-catenin* mRNA levels in the SAMD4B immunoprecipitates versus control IgG immunoprecipitates with *β-catenin* gene-specific primers. *β-actin* mRNA served as an internal reference.

### 2.14. Bioinformatics Analysis

The differential expression of *SAMD4B* mRNA was analyzed in breast cancer tissues versus normal breast tissues, as well as among different clinical stages and molecular subtypes of breast cancer. This analysis utilized data from The Cancer Genome Atlas (TCGA) database, accessed through the UALCAN online platform (http://ualcan.path.uab.edu/index.html (accessed on 22 August 2025)).

### 2.15. Statistical Analysis

Statistical analyses were conducted with GraphPad Prism 6.0 software (GraphPad Software, San Diego, CA, USA), and all data are presented as the mean ± standard deviation (SD). Differences between two independent groups were assessed using an unpaired, two-tailed Student′s *t*-test. One-way or Two-way analysis of variance (ANOVA) was applied to evaluate the significance of mean differences across multiple groups. A *p*-value < 0.05 was considered statistically significant.

## 3. Results

### 3.1. SAMD4B Expression Is Upregulated in Breast Cancer

**Figure 1:**
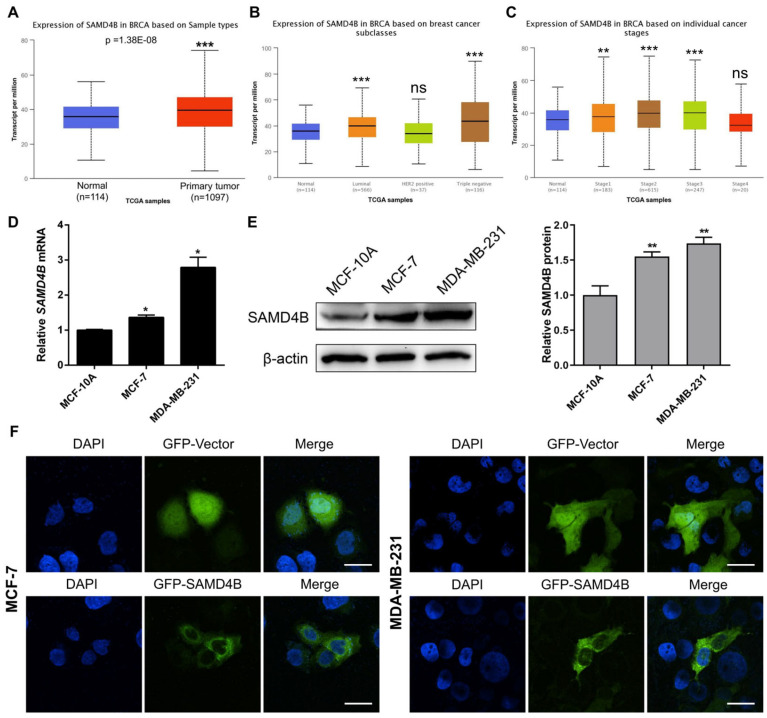
Expression level and subcellular localization of SAMD4B in breast cancer cells. (**A**) The differential expression of *SAMD4B* mRNA in breast cancer tissues and normal breast tissues was assayed by the UALCAN database. (**B**,**C**) Expression patterns of SAMD4B across different molecular subtypes and clinical stages of breast cancer were further analyzed via the UALCAN database. (**D**) *SAMD4B* mRNA expression levels in breast cancer cell lines (MCF-7 and MDA-MB-231) and the normal breast epithelial cell line (MCF-10A) were detected by RT-qPCR analysis. Relative *SAMD4B* mRNA expression was normalized to *GAPDH* mRNA. (**E**) SAMD4B protein expression levels in MCF-10A, MCF-7, and MDA-MB-231 cell lines were determined by Western Blot assay, with β-actin serving as an internal control. Relative SAMD4B protein expression was standardized to β-actin and quantified using ImageJ software. All data are presented as mean ± SD from three independent experiments. * *p* < 0.05, ** *p* < 0.01, and *** *p* < 0.001. “ns” represents no significant difference. (**F**) The subcellular localization of SAMD4B in breast cancer cells was analyzed by immunofluorescence. Briefly, cells were transiently transfected with either GFP empty vector or GFP-SAMD4B recombinant vector. After 24 h of transfection, the subcellular localization of SAMD4B was visualized using a confocal microscope. Scale bar, 10 μm. Original western blot images can be found in Supplementary Materials.

A previous study has identified SAMD4A as a tumor suppressor that is downregulated in breast cancer [26]. In contrast, the expression pattern and functional relevance of its homolog, SAMD4B, in breast cancer remain unknown. To address this, we initially analyzed SAMD4B expression in breast cancer tissues using the TCGA database via the UALCAN tool. The results showed that the *SAMD4B* mRNA expression level was significantly higher in breast cancer tissues than in normal breast tissues (Figure 1A), contrasting with the previously reported low *SAMD4A* expression in breast cancer. Interestingly, *SAMD4B* mRNA expression was significantly upregulated in both luminal and triple-negative breast cancer subtypes (Figure 1B). Furthermore, elevated SAMD4B mRNA levels were observed in clinical stages I, II, and III of breast cancer; however, no significant difference in its expression was detected in stage IV of breast cancer (Figure 1C), indicating that further verification of SAMD4B expression level in clinical breast cancer samples is still needed. To evaluate the potential prognostic relevance of SAMD4B in breast cancer, we performed the Kaplan–Meier survival analysis using multiple databases, including Kaplan–Meier Plotter, UALCAN, GEPIA, and OncoDB. The study revealed no significant difference in overall survival between breast cancer patients with high and low SAMD4B expression (Figure S1).

We next performed RT-qPCR and Western Blot analyses to verify SAMD4B expression in breast cancer cell lines. Compared with the normal breast epithelial cell line MCF-10A, both MCF-7 and MDA-MB-231 breast cancer cell lines exhibited significant upregulation of SAMD4B at the mRNA and protein levels (Figure 1D,E). These results confirm that SAMD4B is highly expressed in breast cancer, suggesting a potential role distinct from that of SAMD4A in breast cancer progression, although additional clinical studies are needed to substantiate these findings.

In addition, we investigated the subcellular localization of SAMD4B in breast cancer cells. Immunofluorescence analysis demonstrated that, relative to the empty vector control group, the green fluorescent protein (GFP) signal driven by SAMD4B was predominantly localized to the cytoplasm in both MCF-7 and MDA-MB-231 cells (Figure 1F). This cytoplasmic localization of SAMD4B may be associated with its previously reported involvement in post-transcriptional regulatory processes, wherein it acts as an RNA-binding protein.

### 3.2. SAMD4B Promotes the Growth and Proliferation of Breast Cancer Cells

**Figure 2:**
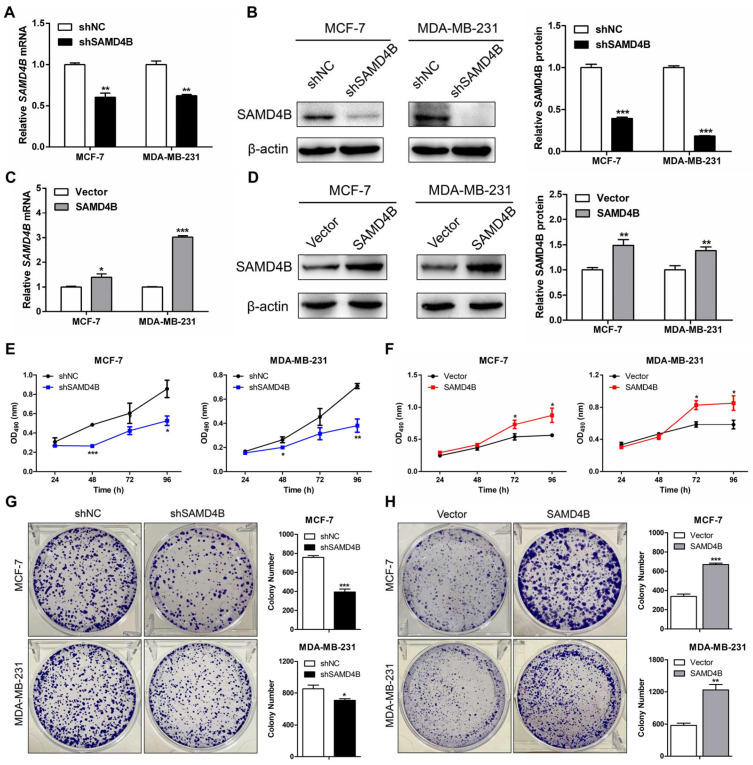
SAMD4B promotes breast cancer cell proliferation. (**A**,**B**) Validation of SAMD4B knockdown efficiency in breast cancer cells by RT-qPCR and Western Blotting, respectively. (**C**,**D**) Validation of SAMD4B overexpression efficiency in breast cancer cells by RT-qPCR and Western Blotting, respectively. (**E**,**F**) Assessment of cell proliferation following SAMD4B knockdown or overexpression by MTT assay. (**G**,**H**) Assessment of cell proliferation following SAMD4B knockdown or overexpression by plate colony formation assay. Data are expressed as mean ± SD (n = 3). Statistical significance is indicated as * *p* < 0.05, ** *p* < 0.01, and *** *p* < 0.001. Abbreviations: shNC, scramble shRNA; shSAMD4B, SAMD4B-targeting shRNA; Vector, empty vector control; SAMD4B, SAMD4B-overexpressing vector. Original western blot images can be found in Supplementary Materials.

To explore the biological function of SAMD4B in breast cancer progression, we first established stable breast cancer cell lines with SAMD4B knockdown or overexpression via lentivirus-mediated infection. The efficiency of SAMD4B knockdown and overexpression in MCF-7 and MDA-MB-231 cells was validated using RT-qPCR (Figure 2A,C) and Western Blot analysis (Figure 2B,D). Compared with their corresponding controls, SAMD4B mRNA and protein expression levels of SAMD4B in MCF-7 and MDA-MB-231 cells were significantly decreased in the knockdown groups and increased in the overexpression groups. These results confirmed the successful construction of stable SAMD4B-knockdown and SAMD4B-overexpressing breast cancer cell lines.

Dysregulated cancer cell proliferation is a hallmark feature of cancer initiation and progression. To delineate the function of SAMD4B in breast cancer cell proliferation, we first utilized the MTT assay to evaluate how SAMD4B modulates the proliferative activity of breast cancer cells. Our data showed that SAMD4B knockdown significantly suppressed the proliferative capacity of breast cancer cells (Figure 2E). Conversely, SAMD4B overexpression markedly enhanced the proliferative potential of these two breast cancer cells (Figure 2F). To further validate these findings, we performed a colony formation assay. Consistent with the MTT results, SAMD4B knockdown reduced the colony-forming ability of breast cancer cells compared with the control group (Figure 2G), whereas SAMD4B overexpression resulted in a significant increase in the colony-forming capacity of breast cancer cells (Figure 2H). Collectively, these complementary experimental data establish SAMD4B as a key promoter in the growth and proliferation of breast cancer cells.

### 3.3. SAMD4B Promotes G1-S Phase Transition of Breast Cancer Cells

**Figure 3:**
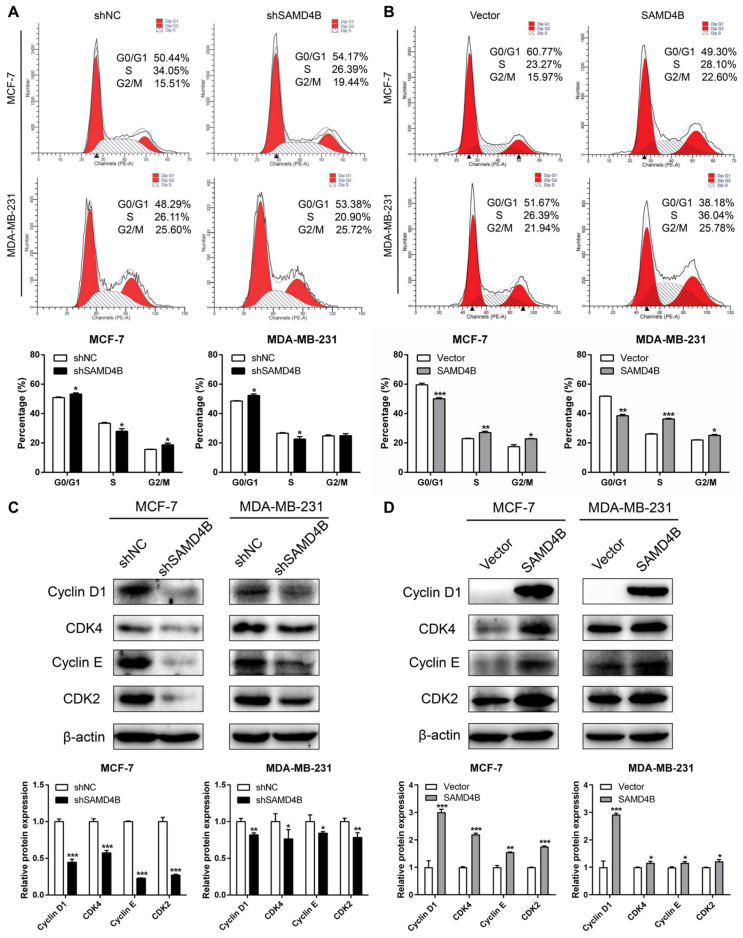
SAMD4B induces G0/G1 phase arrest in breast cancer cells. (**A**,**B**) Flow cytometry analysis was used to assess the impact of SAMD4B knockdown or overexpression on breast cancer cell cycle progression. Upper panels show representative cell cycle profiles, and lower panels show statistical histograms of cell proportions in G0/G1, S, and G2/M phases (n = 3). (**C**,**D**) The protein levels of Cyclin D1, CDK4, Cyclin E, and CDK2 following SAMD4B knockdown or overexpression were detected by Western Blotting, with β-actin used as a loading control. All data are exhibited as mean ±SD, derived from three independent experimental replicates. Statistical significance is indicated as * *p* < 0.05, ** *p* < 0.01, and *** *p* < 0.001. Original western blot images can be found in Supplementary Materials.

Cell cycle dysregulation represents a fundamental hallmark of cancer, frequently contributing to uncontrolled cellular growth and proliferation. To clarify the mechanism underlying SAMD4B-mediated promotion of breast cancer cell growth and proliferation, we investigated its role in regulating cell cycle progression. Flow cytometric analysis revealed that SAMD4B knockdown significantly increased the accumulation of breast cancer cells in the G0/G1 phase, along with a corresponding decrease in the number of cells in the S phase (Figure 3A). In contrast, SAMD4B overexpression reduced the percentage of cells in the G0/G1 phase and elevated the proportion of cells in the S phase (Figure 3B). These data suggest that SAMD4B accelerates the transition of breast cancer cells from the G1 to S phase, consequently facilitating their proliferative capacity.

As is well-established, cyclin-dependent kinases (CDKs) and cyclins are key regulators of the cell cycle control network. CDKs, a family of serine/threonine protein kinases, form functional complexes with cyclins to orchestrate cell cycle progression and modulate gene transcription [35]. The expression dynamics of Cyclin-CDK complexes exhibit characteristic fluctuations across different phases of the cell cycle. We thus investigated whether SAMD4B modulates the expression of G1-phase regulator proteins. Western Blot analysis demonstrated that SAMD4B depletion significantly reduced the expression of G1 phase-specific Cyclin E-CDK2 and Cyclin D1-CDK4 complexes in breast cancer cells (Figure 3C), while SAMD4B overexpression remarkably upregulated these complexes (Figure 3D). Collectively, these findings establish that SAMD4B facilitates breast cancer cell proliferation by upregulating G1-phase Cyclin-CDK complexes, thereby accelerating G1-S phase transition and driving cell cycle progression.

### 3.4. SAMD4B Affects Breast Cancer Cell Cycle Through Regulating p53 Expression

**Figure 4:**
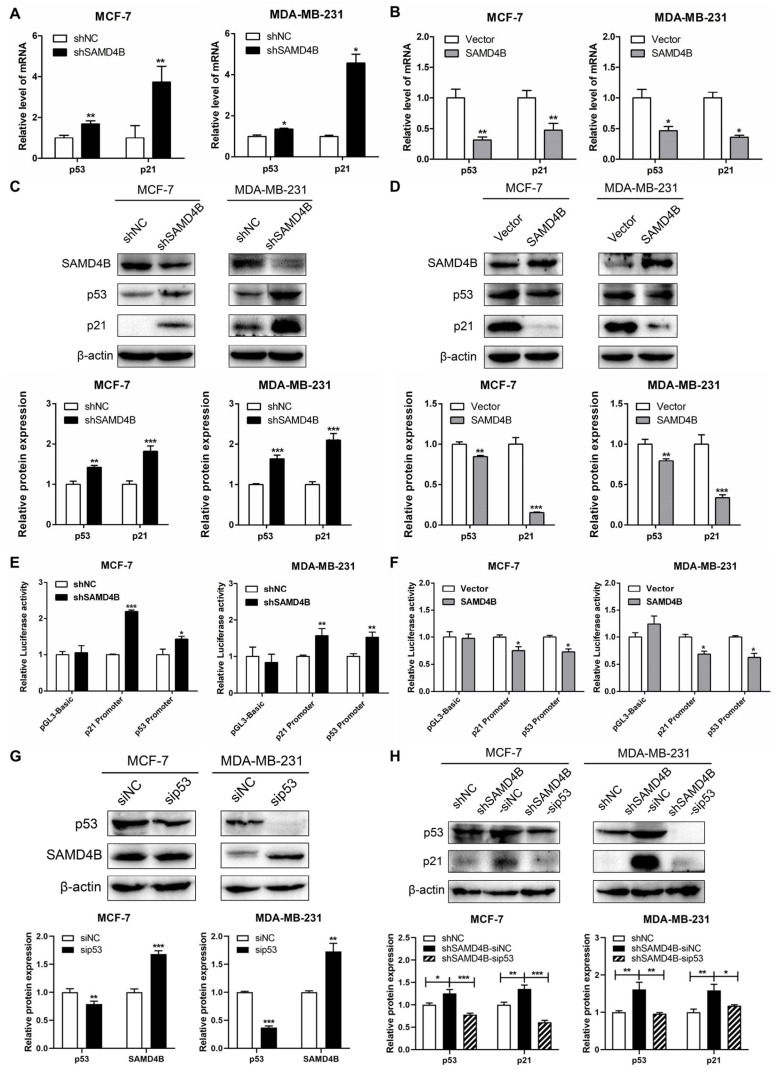
SAMD4B negatively regulates the expression of p53. (**A**,**B**) RT-qPCR analysis was performed to determine *the levels of p53 and p21 mRNA* in breast cancer cells with SAMD4B knockdown or overexpression. (**C**,**D**) Western Blot analysis was employed to detect p53 and p21 protein levels in breast cancer cells with SAMD4B knockdown or overexpression. (**E**,**F**) Dual-luciferase reporter gene assays were performed to evaluate the impact of SAMD4B knockdown or overexpression on the promoter activities of p53 and p21. The relative luciferase activities of the p53 and p21 promoters in SAMD4B-silenced or -overexpressed cells were normalized to those in their respective control cells. (**G**) The impact of p53 knockdown via siRNA transfection on SAMD4B protein expression was analyzed in MCF-7 and MDA-MB-231 breast cancer cells. (**H**) The impact of combined knockdown of SAMD4B and p53 on the protein levels of p21 and p53 was determined. All data are presented as mean ±SD from three biological replicates. Statistical significance is indicated as * *p* < 0.05, ** *p* < 0.01, and *** *p* < 0.001. Original western blot images can be found in Supplementary Materials.

SAMD4B facilitates the G1-to-S phase transition in the breast cancer cell cycle and profoundly modulates the protein expression of G1 phase-specific cyclins and CDKs. As extensively documented, p21 acts as a key negative regulator of the G1-S transition and a critical inhibitor of CDKs. Moreover, p21 is one of the most important transcriptional targets of the tumor suppressor p53, with its expression directly activated by p53. Importantly, the regulatory role of p53 in cell cycle control is predominantly mediated through the modulation of p21 transcription [36,37]. Given this regulatory cascade, we sought to investigate whether SAMD4B exerts its cell cycle effects by influencing the p53-p21 axis. RT-qPCR and Western Blot analyses indicated that SAMD4B knockdown increased the expression of p53 and p21 (Figure 4A,C). By contrast, SAMD4B overexpression led to a substantial reduction in both mRNA and protein levels of p53 and p21 (Figure 4B,D). These findings initially suggested a potential regulatory link between SAMD4B and the p53-p21 pathway. To further delineate the molecular mechanism underlying SAMD4B-mediated regulation of p53 and p21 expression, we performed dual-luciferase reporter assays to evaluate the impact of SAMD4B on p53 and p21 promoter activity. The results showed that SAMD4B knockdown significantly enhanced luciferase activity driven by both the p53 and p21 promoters, whereas SAMD4B overexpression was accompanied by a significant suppression of p53 and p21 promoter activities (Figure 4E,F). These data confirm that SAMD4B modulates p53 and p21 expression primarily at the transcriptional level, thereby providing a mechanistic basis for its role in promoting the G1-S phase progression in breast cancer cells.

To better elucidate how SAMD4B modulates cell cycle progression in breast cancer, we focused on p53, a key regulator in this biological process. We utilized siRNA-mediated p53 knockdown to evaluate its potential compensatory role after SAMD4B depletion. The results of Western Blot analysis showed that p53 knockdown increased the expression of SAMD4B in both MCF-7 and MDA-MB-231 breast cancer cells (Figure 4G). Conversely, SAMD4B knockdown significantly elevated p53 protein level in these cell lines (Figure 4C,H), suggesting that SAMD4B negatively regulates p53 expression. Notably, simultaneous knockdown of SAMD4B and p53 in breast cancer cells also led to a pronounced decrease in p21 protein expression (Figure 4H), which is consistent with p21 being a well-known downstream transcriptional target of p53. Collectively, our findings demonstrate that SAMD4B modulates cell cycle progression in breast cancer through the suppression of p53 expression.

### 3.5. SAMD4B Promotes Migration and Invasion of Breast Cancer Cells Through Inducing EMT

**Figure 5:**
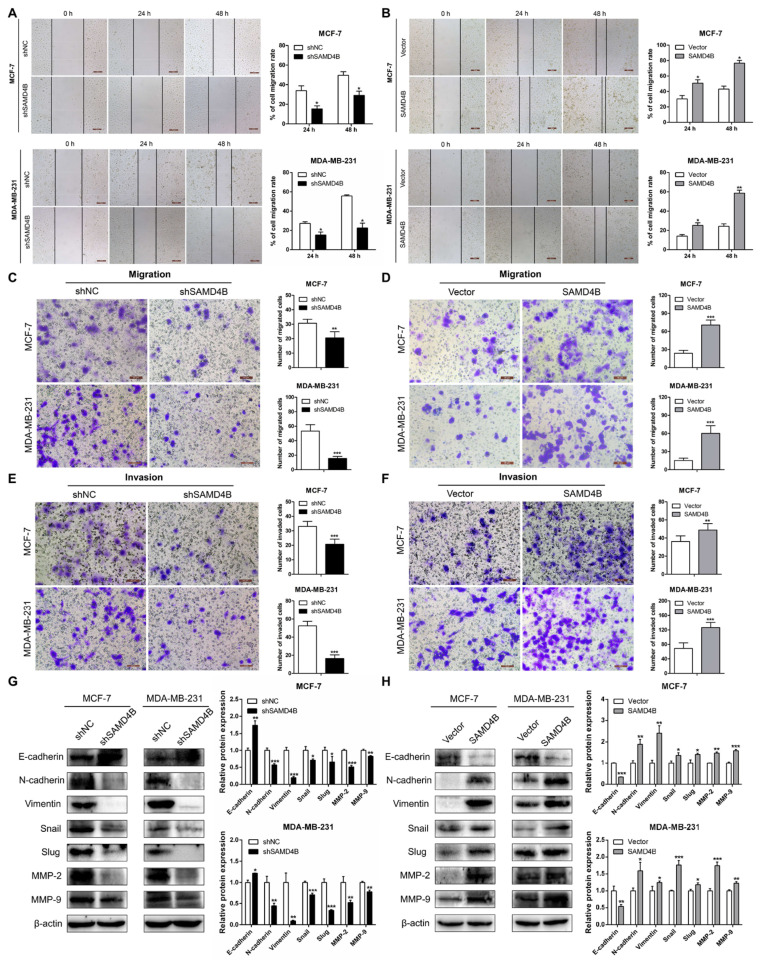
SAMD4B promotes the migration, invasion, and EMT in breast cancer cells. (**A**,**B**) Wound healing assays were conducted to assess the migratory capacity of breast cancer cells with SAMD4B knockdown or overexpression. Representative images were shown at 0, 24, and 48 h (left panels), and the relative cell migration rate at 24 h and 48 h was calculated by normalizing to the baseline at 0 h (right panels). (**C**,**D**) Transwell migration assays were conducted to evaluate the migratory capacity of SAMD4B-silenced or SAMD4B-overexpressing breast cancer cells. Left panels show representative images of migrated cells, while the right panels present quantitative statistical analysis of migrated cells. (**E**,**F**) Transwell Matrigel invasion assays were employed to detect the invasive potential of SAMD4B-silenced or SAMD4B-overexpressing breast cancer cells. Representative images of invaded cells are displayed in the left panels, and quantitative analyses are shown in the right panels. Migrated and invaded cells were quantified in ten random microscopic fields. Scale bar, 50 μm. (**G**,**H**) Western Blot analysis was used to measure the effects of SAMD4B knockdown or overexpression on the protein expression of EMT-related markers (E-cadherin, N-cadherin, Vimentin, Snail, and Slug), and matrix metalloproteinases (MMP-2, MMP-9) in breast cancer cells. β-actin served as the internal loading control. The relative quantification of protein expression was exhibited as mean ± SD from three independent experiments. Statistical significance is indicated as * *p* < 0.05, ** *p* < 0.01, and *** *p* < 0.001. Original western blot images can be found in Supplementary Materials.

To assess SAMD4B’s role in breast cancer metastatic progression, we first conducted wound healing assays. Our results revealed that SAMD4B knockdown significantly delayed the wound closure in both MCF-7 and MDA-MB-231 cells, as reflected by larger residual scratch areas at both 24 h and 48 h compared to controls (Figure 5A). Conversely, SAMD4B overexpression accelerated wound closure, yielding smaller scratch areas compared to control cells in both cell lines (Figure 5B), suggesting that SAMD4B promotes migratory capacity in breast cancer cells. To validate these findings, we conducted Transwell migration assays. Consistent with wound healing results, SAMD4B knockdown significantly reduced the number of migrated cells, while SAMD4B overexpression led to a marked increase in migrated cells relative to controls (Figure 5C,D). We further evaluated the effect of SAMD4B on the invasive capacity of breast cancer cells using Transwell Matrigel assays. The results demonstrated that SAMD4B knockdown substantially suppressed the invasiveness of breast cancer cells, whereas SAMD4B overexpression significantly promoted this phenotype (Figure 5E,F). These collective data identify SAMD4B as a pro-metastatic oncogene that promotes the migration and invasion of breast cancer cells.

It is well established that epithelial–mesenchymal transition (EMT) plays a critical role in tumor initiation, progression, and metastasis [38]. To determine whether the promoting effects of SAMD4B on breast cancer cell migration and invasion are mediated by EMT, we detected the expression changes in EMT-related markers in breast cancer cells upon SAMD4B manipulation. Western Blot analysis revealed that SAMD4B knockdown significantly upregulated the expression of E-cadherin while downregulating the protein levels of N-cadherin, Vimentin, Snail, and Slug (Figure 5G). In contrast, SAMD4B overexpression exhibited the opposite expression pattern of these EMT-related markers (Figure 5H), confirming that SAMD4B promotes the migration and invasion of breast cancer cells through the induction of EMT. Additionally, considering the critical role of matrix metalloproteinases (MMPs) in tumor metastasis, we further examined the effect of SAMD4B on the expression of MMP-2 and MMP-9. Western Blot analysis revealed that SAMD4B knockdown led to a significant decrease in the protein expression of MMP-2 and MMP-9, whereas SAMD4B overexpression increased their expression (Figure 5G,H). Taken together, our data demonstrate that SAMD4B enhances the aggressive phenotype of breast cancer cells by inducing EMT and upregulating MMPs, thereby promoting breast cancer cell proliferation and metastasis.

### 3.6. SAMD4B Activates the Wnt/β-Catenin Pathway in Breast Cancer Cells

**Figure 6:**
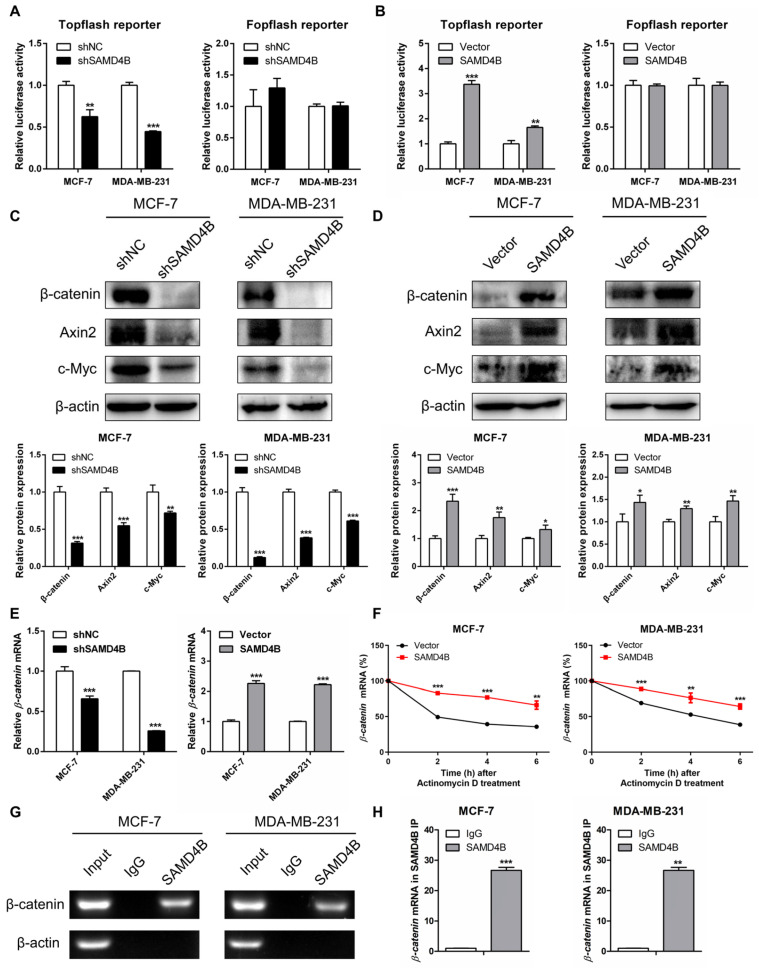
SAMD4B activates the Wnt/β-catenin signaling pathway in breast cancer cells. (**A**,**B**) Topflash/Fopflash dual-luciferase reporter assays were conducted to evaluate the impact of SAMD4B knockdown or overexpression on Wnt/β-catenin pathway activity. Topflash contains functional β-catenin/TCF binding sites (to measure pathway activity), while Fopflash (with mutated binding sites) served as a negative control. (**C**,**D**) Western Blot analysis was used to detect the protein levels of Wnt/β-catenin pathway-related molecules, including β-catenin, c-Myc, and Axin2, in breast cancer cells with SAMD4B knockdown or overexpression. β-actin served as an internal loading control to ensure equal protein loading. (**E**) The effects of SAMD4B knockdown or overexpression on *β-catenin* mRNA expression level were determined by RT-qPCR. The relative *β-catenin* mRNA level was normalized to the control group. (**F**) The impact of SAMD4B overexpression on *β-catenin* mRNA stability. Briefly, SAMD4B-overexpressing breast cancer cells were treated with Actinomycin D at different time points, and *β-catenin* mRNA abundance was analyzed by RT-qPCR at 0, 2, 4, and 6 h. The *β-catenin* mRNA level at 0 h was set as 100% to calculate the relative stability over time. (**G**) RNA immunoprecipitation (RIP) assays were employed to verify the binding of the SAMD4B protein to the *β-catenin* mRNA. MCF-7 and MDA-MB-231 breast cancer cells were transiently transfected with Flag-tagged SAMD4B constructs for 48 h. Cell lysates were immunoprecipitated overnight with anti-Flag antibody (to pull down *SAMD4B*-bound RNA) or anti-IgG antibody (negative control). The co-immunoprecipitated *β-catenin* mRNA transcript was detected by semi-quantitative RT-PCR and visualized via agarose gel electrophoresis. (**H**) The abundance of *β-catenin* mRNA transcript in RIP immunoprecipitates was further quantified by RT-qPCR. All data are exhibited as mean ± SD from three biological replicates. Statistical significance is indicated as * *p* < 0.05, ** *p* < 0.01, and *** *p* < 0.001. Original western blot images can be found in Supplementary Materials.

EMT is a complex biological process orchestrated by numerous regulatory factors and signaling pathways. Among these, the regulatory crosstalk between the Wnt/β-catenin pathway and EMT, in particular, has been extensively documented. Aberrant activation of the Wnt/β-catenin pathway is frequently observed in various human cancers, where it plays a critical role in driving tumor initiation, metastasis, and EMT [33,39]. To dissect the molecular mechanisms underlying SAMD4B-mediated EMT, we explored the regulatory relationship between SAMD4B and the Wnt/β-catenin pathway. Using the Topflash/Fopflash dual-luciferase reporter assay—a specific tool for monitoring Wnt/β-catenin pathway activity—we found that SAMD4B knockdown significantly reduced Topflash reporter activity in both MCF-7 and MDA-MB-231 breast cancer cells, whereas SAMD4B overexpression markedly enhanced Topflash reporter activity. However, Fopflash reporter activity remained unchanged regardless of SAMD4B knockdown or overexpression in either cell line (Figure 6A,B). These results preliminarily demonstrate that SAMD4B enhances the transcriptional activity of TCF/LEF, the key downstream effector of the Wnt/β-catenin pathway, suggesting that SAMD4B activates this pathway in breast cancer cells. To further validate this finding, we examined the protein expression of β-catenin, the central component of the Wnt/β-catenin pathway, as well as its downstream targets Cyclin D1, c-Myc, and Axin2. As shown in Figure 3C,D and Figure 6C,D, SAMD4B knockdown led to a significant downregulation of the protein levels of β-catenin, Axin2, c-Myc, and Cyclin D1, whereas SAMD4B overexpression caused a substantial upregulation of these proteins. Together, these data further confirm that SAMD4B activates the Wnt/β-catenin pathway in breast cancer cells.

To uncover the molecular mechanism through which SAMD4B activates the Wnt/β-catenin signaling pathway in breast cancer, we next investigated its regulatory effect on the mRNA expression of *β-catenin*, a central effector of the pathway. RT-qPCR analysis results demonstrated that knockdown of SAMD4B significantly downregulated *β-catenin* mRNA level, whereas its overexpression markedly upregulated *β-catenin* mRNA expression in breast cancer cells (Figure 6E). These transcriptional changes correlate with corresponding alterations in β-catenin protein level observed following SAMD4B knockdown and overexpression, suggesting a potential link between SAMD4B and *β-catenin* mRNA regulation. To further elucidate how SAMD4B modulates the *β-catenin* mRNA level, we then evaluated the impact of SAMD4B overexpression on the stability of *β-catenin* mRNA. Indeed, in SAMD4B-overexpressing cells, *β-catenin* mRNA exhibited a significantly longer half-life than in control cells (Figure 6F), which can directly explain the elevated *β-catenin* mRNA level induced by SAMD4B overexpression. This prompted the hypothesis that SAMD4B may upregulate β-catenin expression by stabilizing its transcript. To validate this hypothesis, we carried out RNA immunoprecipitation (RIP) assays to examine whether the SAMD4B protein directly interacts with *β-catenin* mRNA. Our results revealed that the *β-catenin* transcript was significantly enriched in SAMD4B-immunoprecipitates compared to control IgG-immunoprecipitates in both MCF-7 and MDA-MB-231 breast cancer cells (Figure 6G,H), demonstrating a specific physical interaction between SAMD4B protein and *β-catenin* mRNA. Collectively, these findings indicate that SAMD4B directly binds to *β-catenin* mRNA, enhances its stability, and thereby upregulates β-catenin expression. This regulatory cascade ultimately triggers the activation of the Wnt/β-catenin pathway in breast cancer cells.

### 3.7. Inhibition of Wnt/β-Catenin Pathway Reverses the Promoting Effects of SAMD4B Overexpression on Breast Cancer Progression

**Figure 7:**
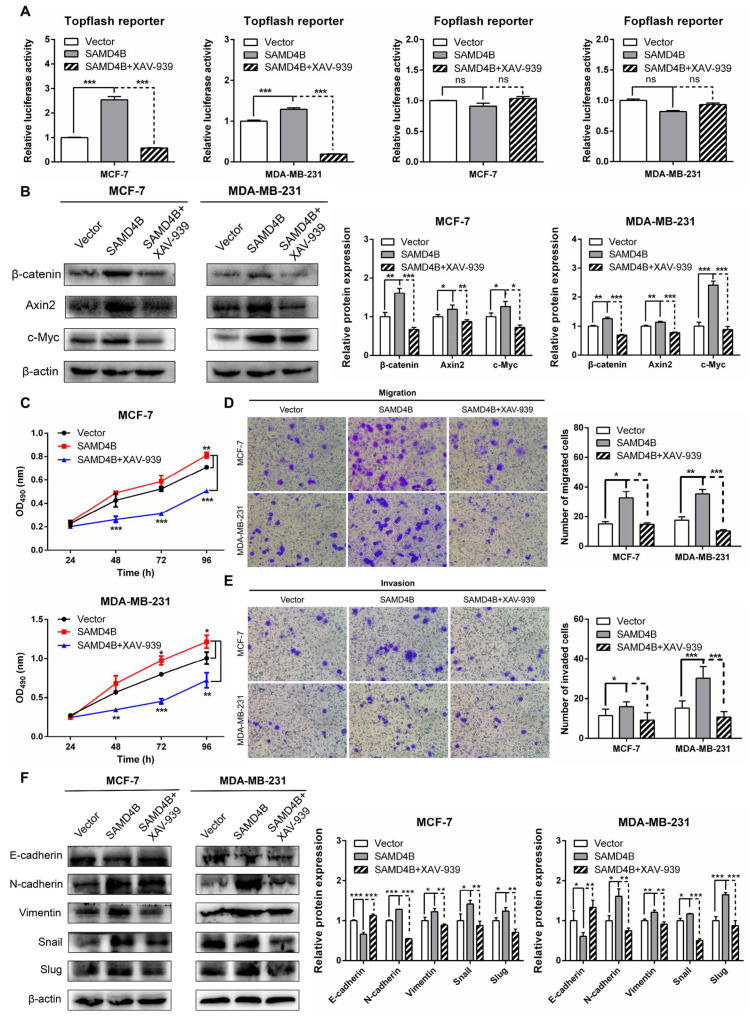
The reversing effects of Wnt/β-catenin pathway inhibitor XAV-939 on SAMD4B overexpression-induced breast cancer cell growth and metastasis. (**A**) Dual-luciferase reporter assay was conducted to determine the Topflash and Fopflash luciferase activities in SAMD4B-overexpressing breast cancer cells treated with 10 μM XAV-939. (**B**) Western Blot assay was employed to detect the expression changes in Wnt/β-catenin pathway-associated proteins (β-catenin, Axin2, and c-Myc) in SAMD4B-overexpressing breast cancer cells following XAV-939 treatment. (**C**) The effect of XAV-939 treatment on the proliferation of SAMD4B-overexpressing breast cancer cells was evaluated via MTT assay. (**D**,**E**) Effects of XAV-939 treatment on migration and invasion of SAMD4B-overexpressing breast cancer cells were examined using Transwell migration and Matrigel invasion assays, respectively. (**F**) Expression changes in EMT-related markers, including E-cadherin, N-cadherin, Vimentin, Snail, and Slug, in SAMD4B-overexpressing breast cancer cells treated with XAV-939. Protein expression levels were quantified and normalized relative to the control group. The data are expressed as mean ± SD from three independent experiments (n = 3). Statistical significance is indicated as * *p* < 0.05, ** *p* < 0.01, and *** *p* < 0.001. “ns” represents no significant difference. Original western blot images can be found in Supplementary Materials.

To confirm whether SAMD4B promotes the growth and metastasis of breast cancer cells by directly activating the Wnt/β-catenin pathway, we treated SAMD4B-overexpressing breast cancer cells with XAV-939, a specific inhibitor of the Wnt/β-catenin pathway and systematically evaluated the regulatory effects of this intervention on breast cancer cell proliferation and metastatic potential. We first validated the capacity of XAV-939 to block SAMD4B overexpression-induced activation of the Wnt/β-catenin pathway in breast cancer cells. Topflash/Fopflash dual-luciferase reporter assays demonstrated that compared to the untreated SAMD4B-overexpressing group, XAV-939 treatment significantly reduced Topflash luciferase activity. In contrast, Fopflash luciferase activity remained unchanged across groups (Figure 7A). These findings confirm that XAV-939 specifically inhibits the transcriptional activity of TCF/LEF and blocks the Wnt/β-catenin pathway. To further corroborate this observation, we performed Western Blot analysis to examine the protein levels of Wnt/β-catenin pathway-related molecules. As shown in Figure 7B, compared with the untreated SAMD4B-overexpressing group, XAV-939 treatment caused a marked downregulation of β-catenin, Axin2, and c-Myc protein levels. Collectively, these complementary experimental results strongly support the conclusion that SAMD4B activates the Wnt/β-catenin signaling pathway in breast cancer cells.

We next evaluated the impact of XAV-939-mediated Wnt/β-catenin pathway inhibition on the proliferative and metastatic phenotype of SAMD4B-overexpressing breast cancer cells. MTT proliferation assays revealed that XAV-939 treatment significantly attenuated the enhanced proliferative capacity induced by SAMD4B overexpression (Figure 7C). Consistent with these findings, Transwell migration and Matrigel invasion assays revealed that XAV-939 treatment effectively reversed enhanced migratory and invasive capabilities induced by SAMD4B overexpression (Figure 7D,E). To explore the molecular basis underlying these phenotypic changes, we further verified the protein levels of EMT-associated markers in XAV-939-treated SAMD4B-overexpressing cells. Western Blot analysis confirmed that XAV-939 treatment reversed the SAMD4B-induced alterations in EMT-associated marker proteins, including the increase in E-cadherin and decrease in N-cadherin, Vimentin, Snail, and Slug (Figure 7F). These results suggest that XAV-939 abrogates SAMD4B-mediated EMT progression. Taken together with our previous observations, these pharmacological inhibition experiments substantiate that SAMD4B enhances the proliferative and metastatic capacities of breast cancer cells primarily via activation of the Wnt/β-catenin pathway. The fact that XAV-939 reverses all major SAMD4B-induced oncogenic phenotypes further strengthens the conclusion that SAMD4B exerts its function through this signaling axis.

## 4. Discussion

RNA-binding proteins (RBPs) have emerged as a central focus in RNA biology research in recent years, as they play essential roles in the post-transcriptional regulation of gene expression in eukaryotic cells. Accumulating evidence has established that RBPs are closely implicated in cancer initiation and progression [40]. Dysregulation or mutation of RBPs in cancers can modulate target gene expression by regulating mRNA stability and translation, thereby influencing multiple hallmarks of cancer, such as cell proliferation, apoptosis, migration, invasion, metastasis, and angiogenesis [8,41]. The SAMD4 family is a novel class of RBPs, comprising two members, SAMD4A and SAMD4B, both of which have been recognized as new post-transcriptional regulators and translational repressors [14,42,43]. SAMD4 family exerts diverse biological functions through its conserved SAMD domain, a functional RNA-binding module, which can directly interact with a stem-loop structure located in the 3′-UTR of target mRNA [14]. Currently, a multitude of studies have linked SAMD4 family members to cancer pathogenesis. For example, SAMD4A showed elevated expression in topotecan-resistant ovarian cancer cell lines and correlates with tumor chemoresistance [44,45]. SAMD4B is confirmed as a direct target of miR-451, and miR-451 inhibits colorectal cancer cell malignancy by targeting the 3′-UTR of *SAMD4B* mRNA [29]. Additionally, whole-genome sequencing of patients with acute myeloid leukemia has uncovered recurrent mutations in the *SAMD4B* gene [46]. A recent research work demonstrated that SAMD4A expression is downregulated in breast cancer, where it functions as a tumor suppressor to inhibit breast tumor angiogenesis [26]. Nevertheless, the function of SAMD4B in breast cancer is largely unexplored. Therefore, our study is the first to systematically investigate the functional role and specific molecular mechanisms of SAMD4B in breast cancer progression.

**Figure 8:**
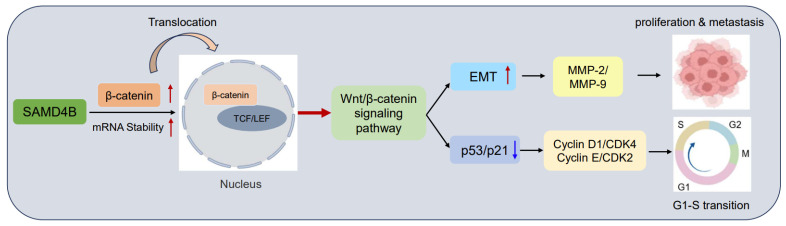
Schematic diagram of the molecular mechanism underlying SAMD4B-mediated promotion of breast cancer progression. This schematic diagram illustrates that SAMD4B facilitates the proliferation and metastasis of breast cancer cells, as well as the G1-to-S phase transition, by activating the Wnt/β-catenin signaling pathway.

In the present study, we demonstrated that, unlike tumor suppressor SAMD4A, SAMD4B was significantly upregulated in breast cancer. Functional assays confirmed the oncogenic role of SAMD4B in breast cancer pathogenesis. Specifically, knockdown of SAMD4B significantly inhibited the proliferation, migration, invasion, EMT, and induced cell cycle arrest of breast cancer cells, while its overexpression robustly promoted these malignant phenotypes. Mechanistically, SAMD4B is found to enhance *β-catenin* mRNA stability, leading to elevated β-catenin expression and consequent activation of the Wnt/β-catenin pathway, as summarized in Figure 8. These results strongly suggest that SAMD4B may function as an oncogene in breast cancer, and its function is in direct opposition to the tumor-suppressive role of SAMD4A [26]. This functional antagonism within the SAMD4 family highlights a key dichotomy in breast cancer progression and reflects evolutionary divergence in the protein family. The homologous proteins assume oncogenic and tumor-suppressive roles, respectively, in the tumor microenvironment due to differences in their protein sequences or molecular regulatory mechanisms.

Previous studies reported that SAMD4B overexpression inhibited the transcriptional activities of AP-1, p53 and p21 [28]. Given the well-documented role of p53 in regulating the tumor cell cycle and apoptosis [47], the precise relationship between SAMD4B and breast cancer cell cycle progression remained unclear. Our study demonstrates that SAMD4B knockdown induced G0/G1 phase arrest, accompanied by reduced expression of G1-phase regulatory complexes Cyclin E-CDK2 and Cyclin D1-CDK4. Conversely, overexpression of SAMD4B upregulated these two complexes, indicating its role in promoting G1-S phase transition. Mechanistically, SAMD4B overexpression decreased both mRNA and protein levels of p53 and p21 by inhibiting their promoter activities, which is consistent with previous reports indicating that SAMD4B inhibits the transcriptional activity of p53 [28]. Thus, SAMD4B transcriptionally represses p53 and p21 to facilitate G1-S transition. Importantly, co-knockdown of p53 in SAMD4B-silenced cells abolished the induction of p21, indicating that the upregulation of p21 caused by SAMD4B depletion is dependent on p53. These results demonstrate that SAMD4B transcriptionally represses p53 and p21 to facilitate G1–S transition, and establish p53 as a central regulator of SAMD4B-mediated cell cycle regulation in breast cancer.

Invasion and metastasis are the hallmark features of malignant tumors and constitute the leading cause of mortality in breast cancer patients. However, the biological function of SAMD4B in tumor metastasis remains unclear. To address this, we conducted a series of in vitro functional assays and demonstrated that SAMD4B overexpression enhanced the proliferation, migration, and invasion capacities, as evidenced by MTT, colony formation, wound healing, and Transwell experiments. In contrast, SAMD4B knockdown attenuated these malignant phenotypes. Our study is the first to establish an oncogenic role for SAMD4B in breast cancer, providing new mechanistic insights into breast cancer progression. A notable limitation of the current study lacks sufficient in vivo validation using animal models to verify the oncogenic function of SAMD4B in promoting breast cancer metastasis. Although our in vitro results clearly indicate that SAMD4B enhances metastatic phenotypes of breast cancer cells, these findings cannot reflect the complexity of the tumor microenvironment in vivo. Therefore, further in vivo investigations are warranted to validate the pro-metastatic function of SAMD4B in breast cancer.

Although we have confirmed the upregulated expression of SAMD4B and its oncogenic role in breast cancer progression, there was no significant difference in the overall survival of breast cancer patients stratified by SAMD4B expression. This discrepancy, however, does not negate its oncogenic function and may be explained by several factors: (i) SAMD4B may possess a subtype-specific function, necessitating prognostic evaluation within distinct molecular subtypes; (ii) SAMD4B may synergistically interact with other RNA-binding proteins to modulate oncogenic pathways, such that its prognostic impact becomes evident only when combined with the expression of these co-regulators; and (iii) SAMD4B may primarily drive early-stage tumor initiation and invasion rather than long-term survival, which aligns with its role in regulating EMT.

EMT is the primary step in tumor invasion and metastasis. A key characteristic of cancer cells undergoing EMT is the acquisition of malignant invasive and metastatic capabilities, accompanied by alterations in the expression of EMT-related genes [48]. We therefore investigated the regulatory role of SAMD4B in EMT of breast cancer cells. Our data demonstrated that SAMD4B overexpression downregulated E-cadherin while upregulating N-cadherin, Vimentin, Snail, and Slug, suggesting that SAMD4B promotes EMT in breast cancer cells. It also increased the expression of MMP-2 and MMP-9. In contrast, SAMD4B knockdown exerted opposite effects on these EMT-related markers and MMPs. To our knowledge, this study is the first to document SAMD4B’s role in inducing EMT. Our findings demonstrate that SAMD4B promotes breast cancer cell proliferation and metastasis through the induction of EMT, which provides key insights into the molecular mechanism underlying breast cancer metastasis. These findings highlight that the SAMD4B-EMT axis may represent a potential therapeutic target for breast cancer. Therapeutic strategies could include developing small-molecule inhibitors that specifically target SAMD4B or designing drugs that block the EMT-related signaling pathways.

EMT is regulated by multiple signaling pathways and regulatory factors. In human cancers, aberrant activation of the Wnt/β-catenin pathway is frequently associated with EMT induction, elevated MMP expression, and enhanced metastasis and drug resistance [33,49]. However, whether SAMD4B modulates EMT of breast cancer cells via this pathway remained unknown. Therefore, we explored the correlation between SAMD4B’s oncogenic role and the activation of the Wnt/β-catenin pathway. SAMD4B overexpression increased Topflash luciferase activity and upregulated the expression of β-catenin, Axin2, Cyclin D1, and c-Myc in breast cancer cells. Conversely, SAMD4B knockdown suppressed Topflash luciferase activity and the expression of these target genes. These results suggest that SAMD4B-mediated tumorigenesis and metastasis correlate with Wnt/β-catenin pathway activation. Mechanistically, we confirmed that SAMD4B binds to *β-catenin* mRNA to enhance its stability, thereby upregulating β-catenin expression and promoting its nuclear translocation. More importantly, inhibitor experiments validated this conclusion: treatment with XAV-939 (a specific inhibitor of the Wnt/β-catenin pathway) reversed SAMD4B overexpression-induced increases in TCF/LEF transcriptional activity and the expression of β-catenin, Axin2, and c-Myc. Additionally, XAV-939 abrogated SAMD4B-driven promotion of breast cancer cell proliferation, migration, invasion, and EMT. In conclusion, SAMD4B promotes EMT of breast cancer cells at least partly by activating the Wnt/β-catenin pathway, thereby facilitating tumorigenesis and metastasis. These findings uncover that the SAMD4B-Wnt/β-catenin axis may serve as a potential key mechanism in regulating breast cancer progression.

Taken together, our research identifies that SAMD4B exerts an oncogenic role in promoting breast cancer progression via activating the Wnt/β-catenin signaling pathway, highlighting its potential as a novel therapeutic target for breast cancer. This oncogenic role of SAMD4B is functionally analogous to that of the known scaffolding protein in breast cancer pathogenesis [50,51]. Although our research advances understanding of SAMD4B’s oncogenic role in breast cancer progression, further in vivo investigations are required to clarify its specific molecular mechanisms in tumorigenesis and metastasis. Future studies should include the following: (i) constructing SAMD4B knockdown/overexpression animal models to evaluate its impact on tumor growth, metastasis and Wnt/β-catenin pathway activation; (ii) analyzing SAMD4B expression in clinical breast cancer patient tissues to determine its correlation with survival prognosis; (iii) examining the precise mechanisms through which SAMD4B stabilizes *β-catenin* mRNA to map specific binding sites and identify potential cofactors by RIP-seq or CLIP-seq assays; and (iv) further exploring the interactions between SAMD4B and other EMT-related signaling pathways, along with its regulatory role in the tumor microenvironment. These investigations will lay a solid foundation for precision therapy of breast cancer and inform the development of effective therapeutic strategies.

## 5. Conclusions

In summary, our in vitro findings establish that SAMD4B acts as a novel oncogene in breast cancer. SAMD4B post-transcriptionally upregulates β-catenin expression by enhancing *β-catenin* mRNA stability, thereby activating the Wnt/β-catenin signaling pathway. This pathway activation induces EMT and consequently promotes breast cancer cell proliferation, migration, and invasion. Furthermore, SAMD4B facilitates breast cancer cell cycle progression through the downregulation of p53 and p21, and the concomitant upregulation of Cyclin E-CDK2 and Cyclin D1-CDK4 complexes, thereby driving the G1/S phase transition. Collectively, these findings strongly support the oncogenic role of SAMD4B and validate it as a promising therapeutic target for breast cancer intervention.

## Figures and Tables

**Table 1:** Specific primer sequences employed in qPCR experiments.

Gene	Forward Primer (5ʹ-3ʹ)	Reverse Primer (5ʹ -3ʹ)
*SAMD4B* *p21* *p53* *β-catenin* *GAPDH*	CCTGCCAAATCCACCCTAGC GGCGAGGCCGGGATGAGTTG GAGGTTGGCTCTGACTGTACC AGCTTCCAGACACGCTATCAT CGCTCTCTGCTCCTCCTGTT	TGATCGGGCGTGGTAAAAGC CTGCCGCCGTTTTCGACCCT TCCGTCCCAGTAGATTACCAC CGGTACAACGAGCTGTTTCTAC CCATGGTGTCTGAGCGATGT

## Data Availability

The data presented in this study are available in the article and Supplementary Materials.

## Supplementary Materials

The following supporting information can be downloaded at: https://www.mdpi.com/article/10.3390/biom15101423/s1, Figure S1: Kaplan-Meier analysis of overall survival in breast cancer patients with high or low expression of SAMD4B mRNA expression. (**A**) The Kaplan-Meier Plotter database (https://kmplot.com/analysis/ (accessed on 22 August 2025)) was used to analyze the overall survival in breast cancer patients stratified by SAMD4B expression levels. (**B**) The UALCAN database (http://ualcan.path.uab.edu/index.html (accessed on 22 August 2025)) was applied for analyzing the overall survival in breast cancer patients stratified by SAMD4B expression levels. (**C**) Overall survival in breast cancer patients stratified by SAMD4B expression levels was analyzed using the GEPIA database (http://gepia.cancer-pku.cn/ (accessed on 22 August 2025)). (D) The OncoDB database (https://oncodb.org (accessed on 22 August 2025)) served to analyze overall survival in breast cancer patients stratified by SAMD4B expression levels; Figure S2: Original Western Blot gels corresponding to each figure. (**A**) Original Western Blot gels for Figure 1E. (**B**) Original Western Blot gels for Figure 2B,D. (**C**) Original Western Blot gels for Figure 3C,D. (**D**) Original Western Blot gels for Figure 4C,D,G,H. (**E**) Original Western Blot gels for Figure 5G,H. (**F**) Original Western Blot gels for Figure 6C,D. (**G**) Original Western Blot gels for Figure 7B. (**H**) Original Western Blot gels for Figure 7F.

## Abbreviations

The following abbreviations are used in this manuscript:

SAMD4B	Sterile alpha motif domain-containing protein 4B
EMT	Epithelial–mesenchymal transition
MMP	Matrix metalloproteinase
CDK	Cyclin-dependent kinases
RIP	RNA immunoprecipitation
HBV	Human hepatitis B virus
UTR	Untranslated region
CCR4	Carbon Catabolite Repressor 4
POP2	Poly(A) polymerase-associated protein 2
KDM2B	Lysine (K)-specific demethylase 2B
FGF2	Fibroblast growth factor 2
ATF4	Activating transcription factor 4
hnRNA	Heterogeneous nuclear RNA
BAG2	Bcl2-associated athanogene 2
RACK1	Receptor for activated C kinase 1
