# Supplementary material for: Oncogenic Role of SAMD4B in Breast Cancer Progression by Activating Wnt/β-Catenin Pathway
Source: Biomolecules. 2025 Oct 7;15(10):1423. doi: 10.3390/biom15101423 (PMC12563151; doi:10.3390/biom15101423)
Supplement: Supplementary file 1 [file biomolecules-15-01423-s001.zip › biomolecules-3862913-supplementary.pdf]

Supplementary Materials

Oncogenic Role of SAMD4B in Breast Cancer Progression by Activating Wnt/β-Catenin Pathway

Jia-Hui Li †, Xin-Ya Wang †, Huan-Xi Song, Xiao-Fei Nie and Li-Na Zhang *

Beijing International Science and Technology Cooperation Base of Antivirus Drug, College of Chemistry and Life Science, Beijing University of Technology, Beijing 100124, China; ljhuiii@emails.bjut.edu.cn (J.-H.L.); wangxinya@emails.bjut.edu.cn (X.-Y.W.); songhuanxi@emails.bjut.edu.cn (H.-X.S.); niexiaofei@emails.bjut.edu.cn (X.-F.N.)

* Correspondence: lnzhang@bjut.edu.cn; Tel.: +86-10-6739-6342

† These authors contributed equally to this work.

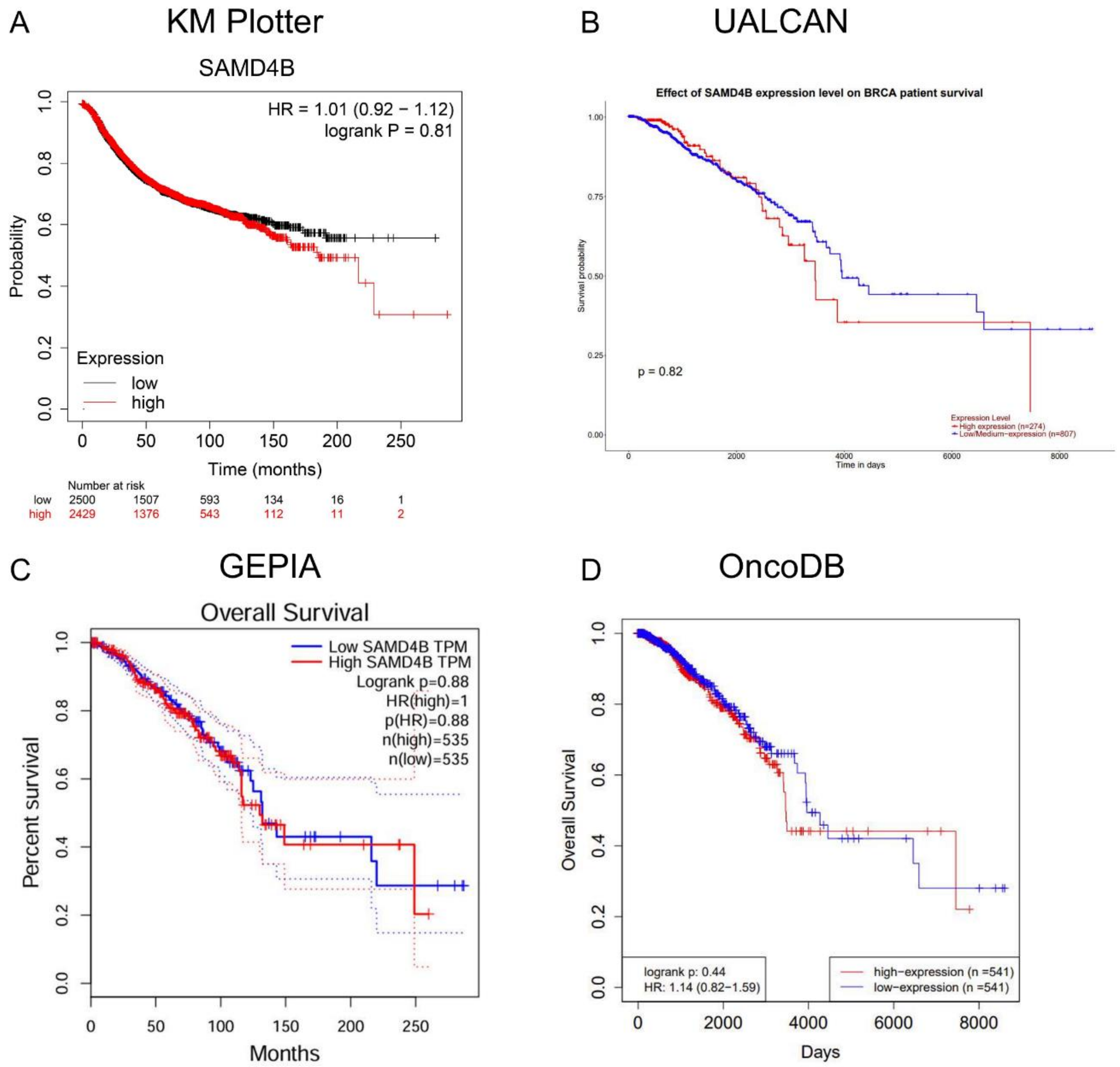

Figure S1. Kaplan-Meier analysis of overall survival in breast cancer patients with high or low expression of *SAMD4B* mRNA expression. (A) The Kaplan-Meier Plotter database (https://kmplot.com/analysis/) was used to analyze the overall survival in breast cancer patients stratified by SAMD4B expression levels. (B) The UALCAN database (http://ualcan.path.uab.edu/index.html) was applied for analyzing the overall survival in breast cancer patients stratified by SAMD4B expression levels. (C) Overall survival in breast cancer patients stratified by SAMD4B expression levels was analyzed using the GEPIA database (http://gepia.cancer-pku.cn/). (D) The OncoDB database (https://oncodb.org) served to analyze overall survival in breast cancer patients stratified by SAMD4B expression levels.

Figure S2, Original Western Blot gels.

(A) Original Western Blot gels for Figure 1E

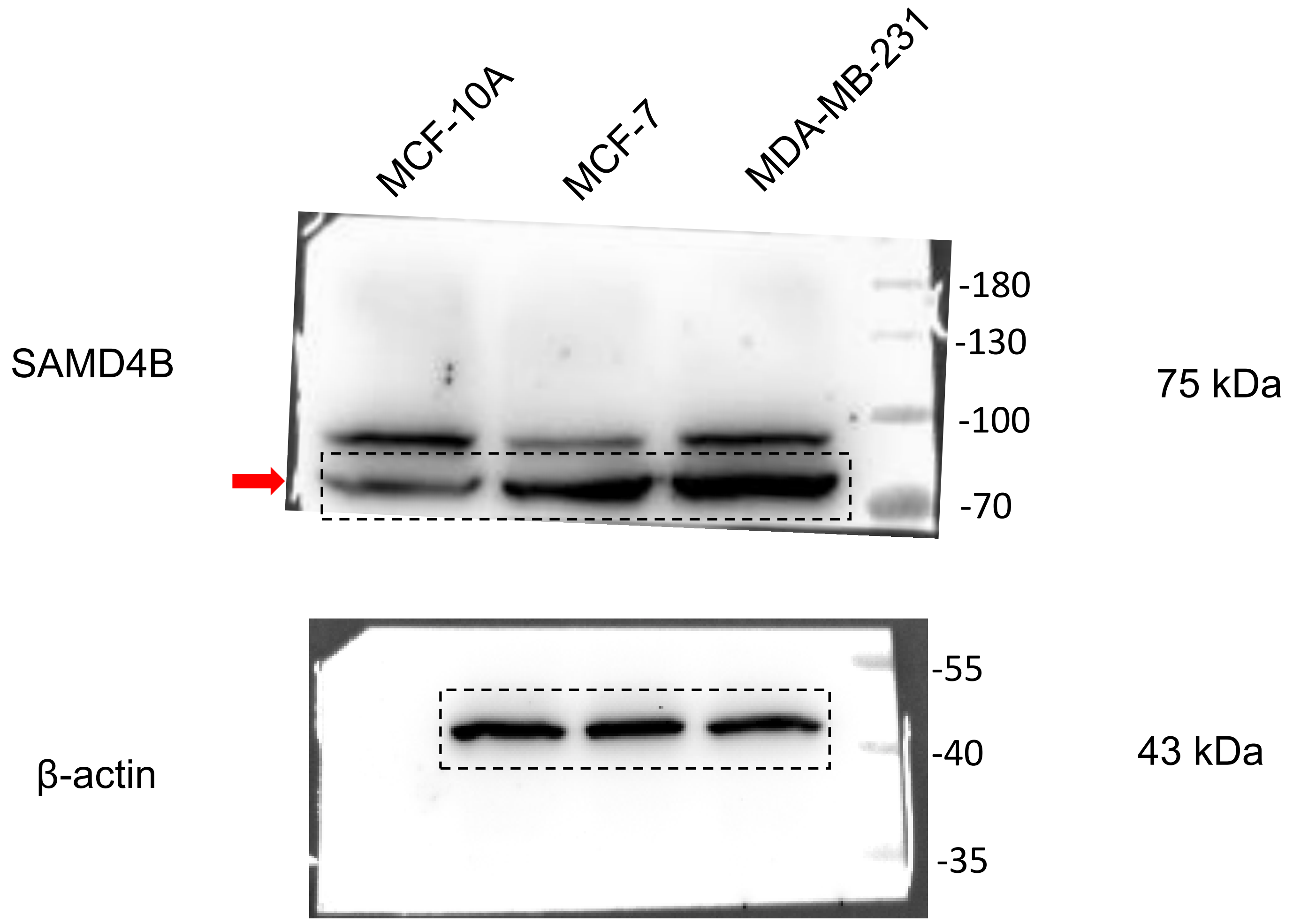

(B) Original Western Blot gels for Figure 2B and 2D

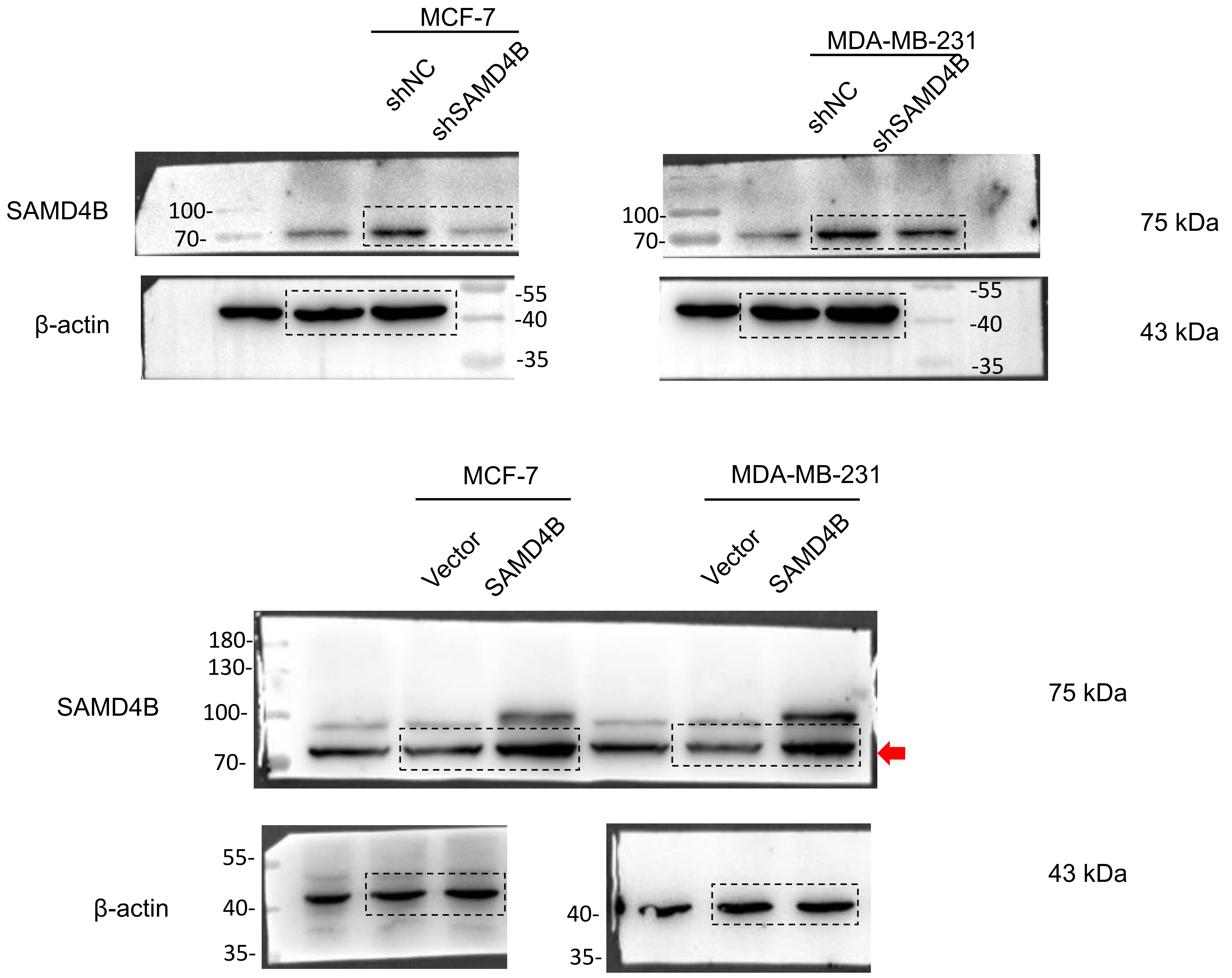

(C) Original Western Blot gels for Figure 3C and 3D

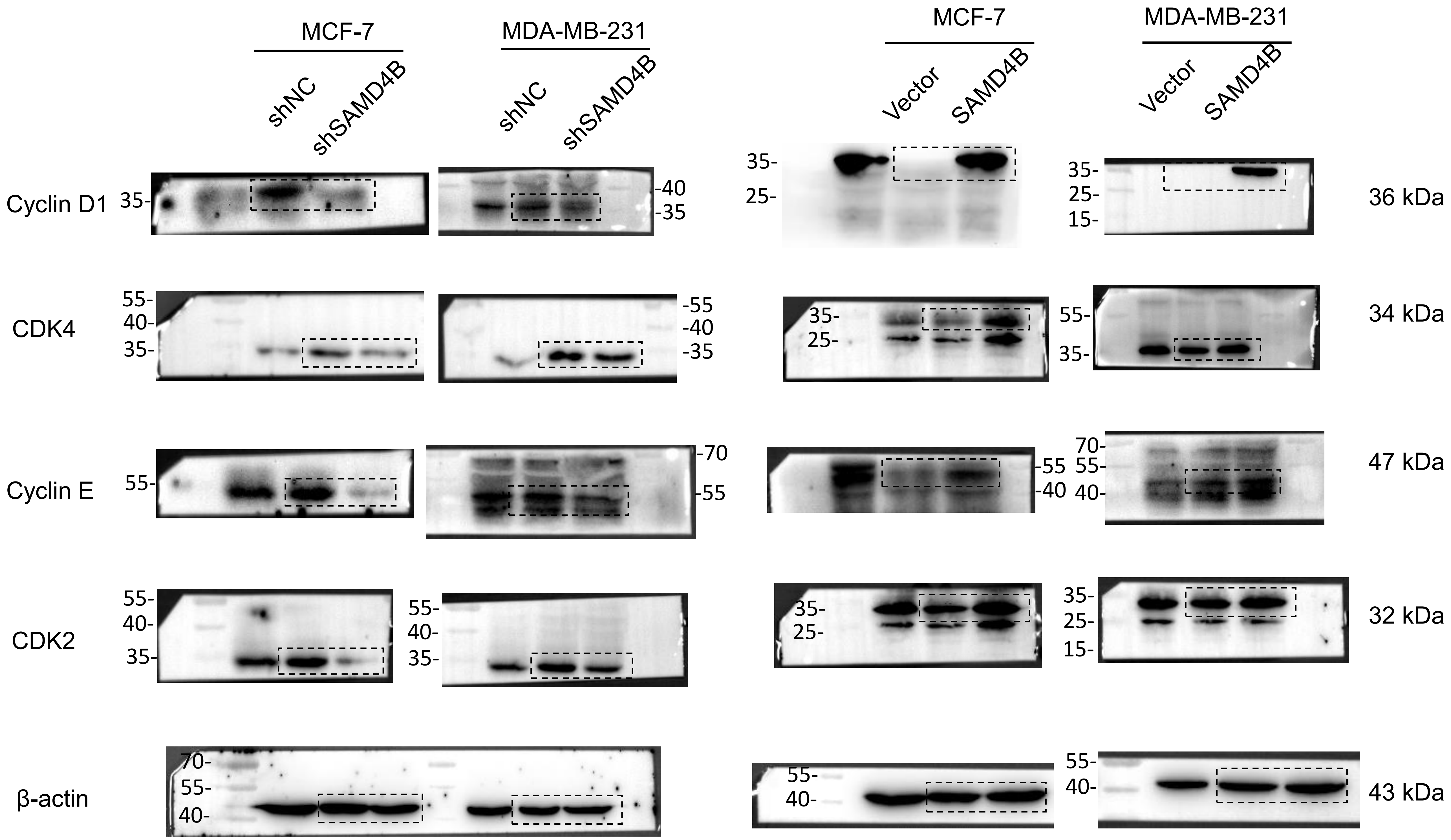

(D) Original Western Blot gels for Figure 4C, 4D, 4G, 4H

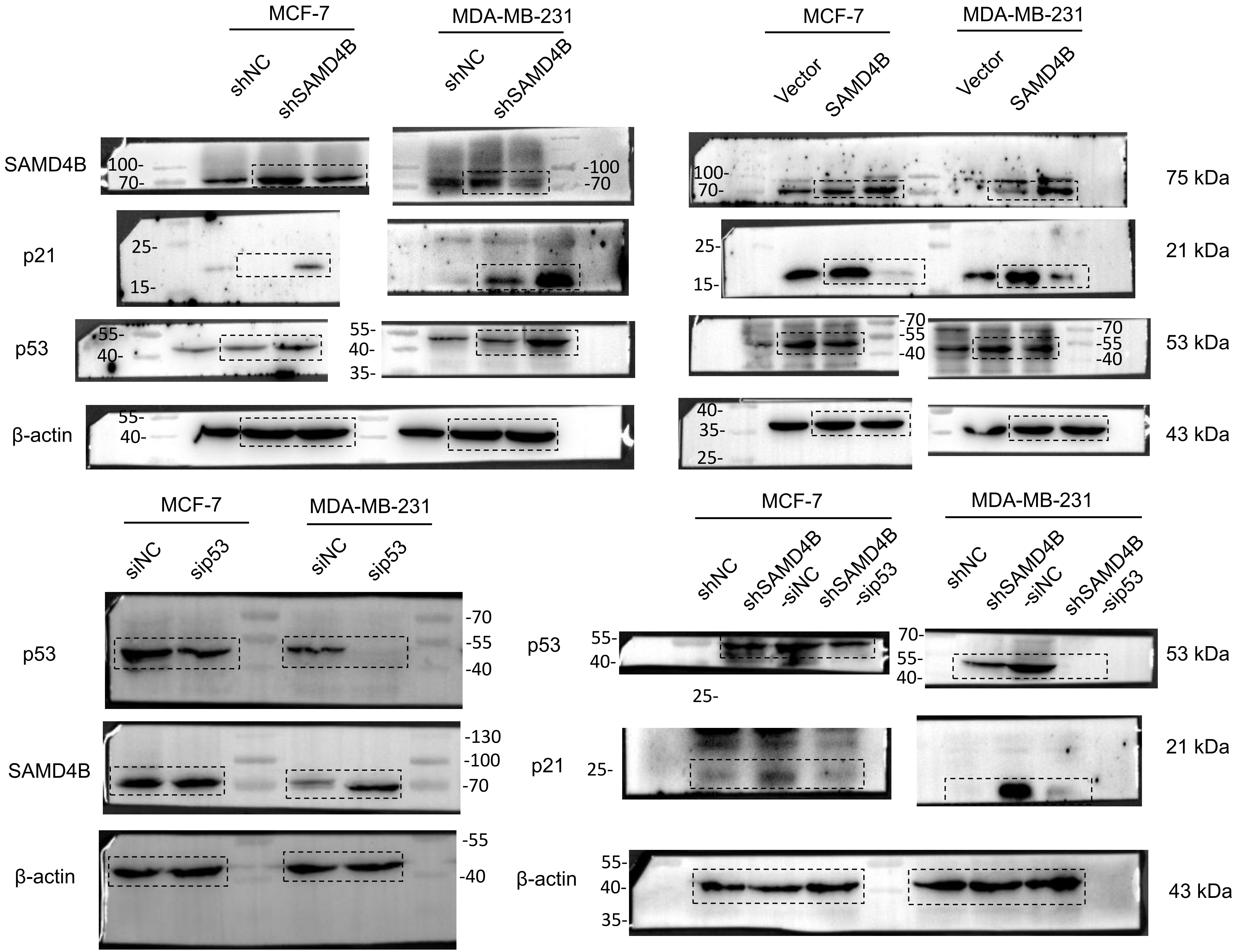

(E) Original Western Blot gels for Figure 5G and 5H

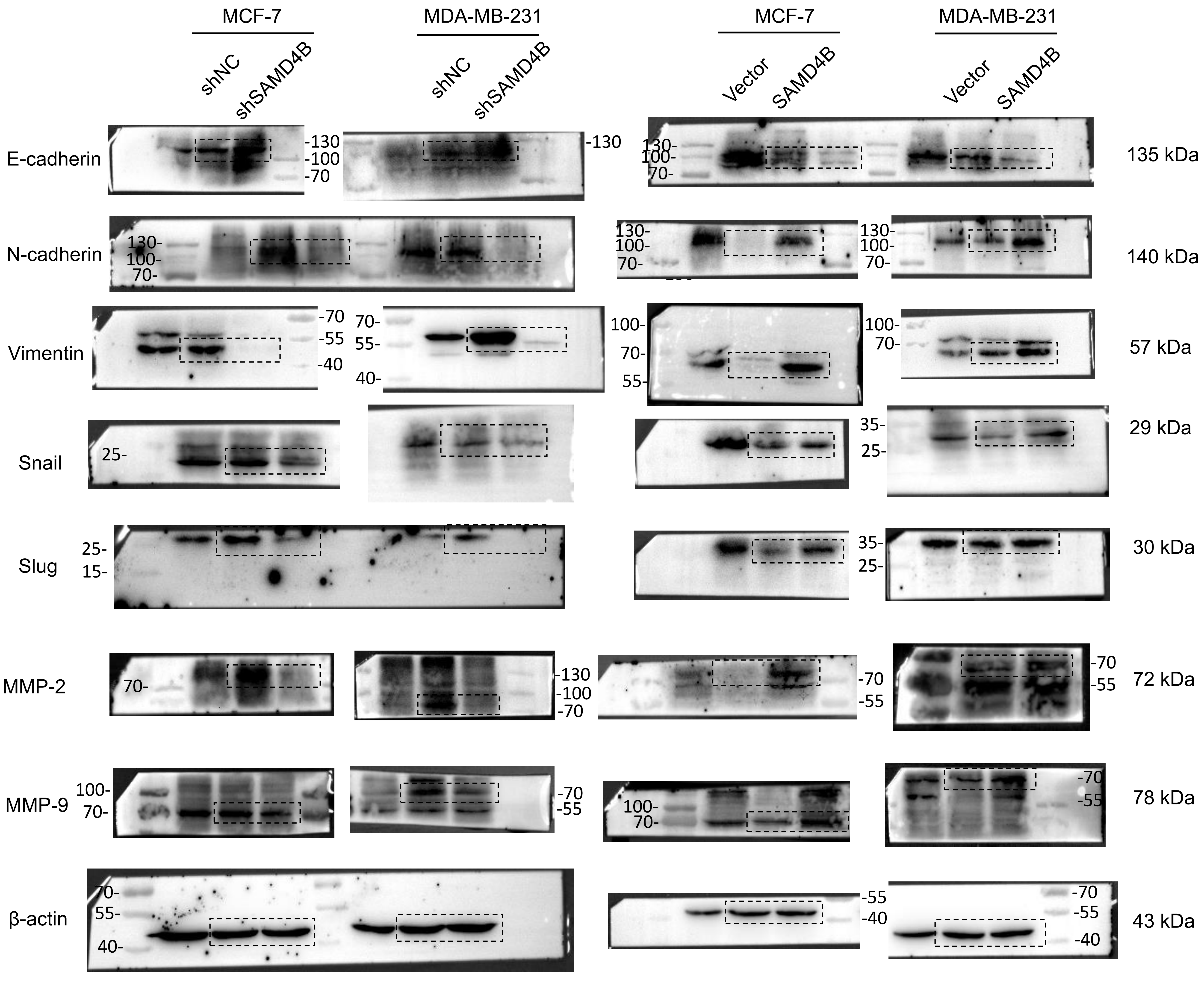

(F) Original Western Blot gels for Figure 6C and 6D

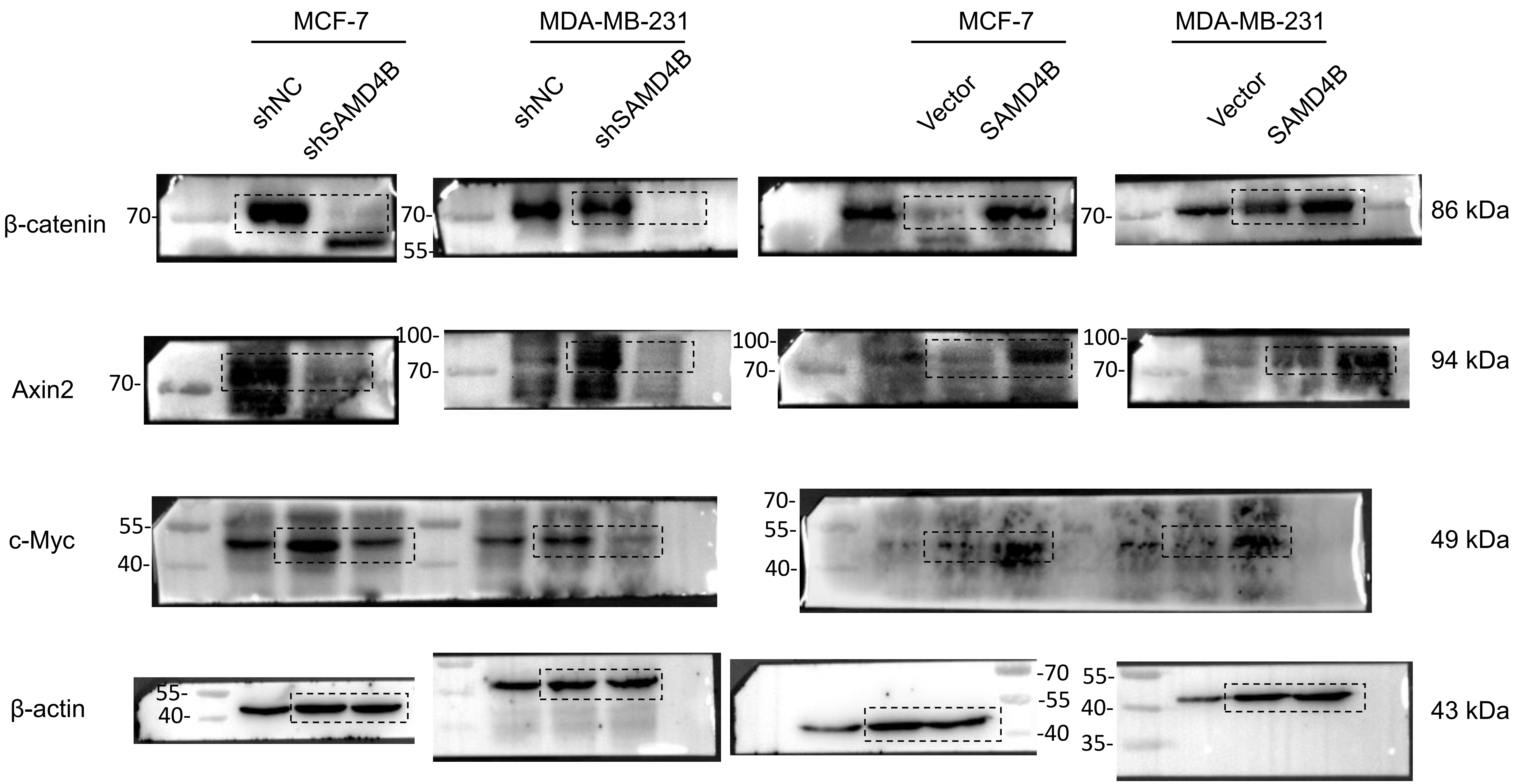

(G) Original Western Blot gels for Figure 7B

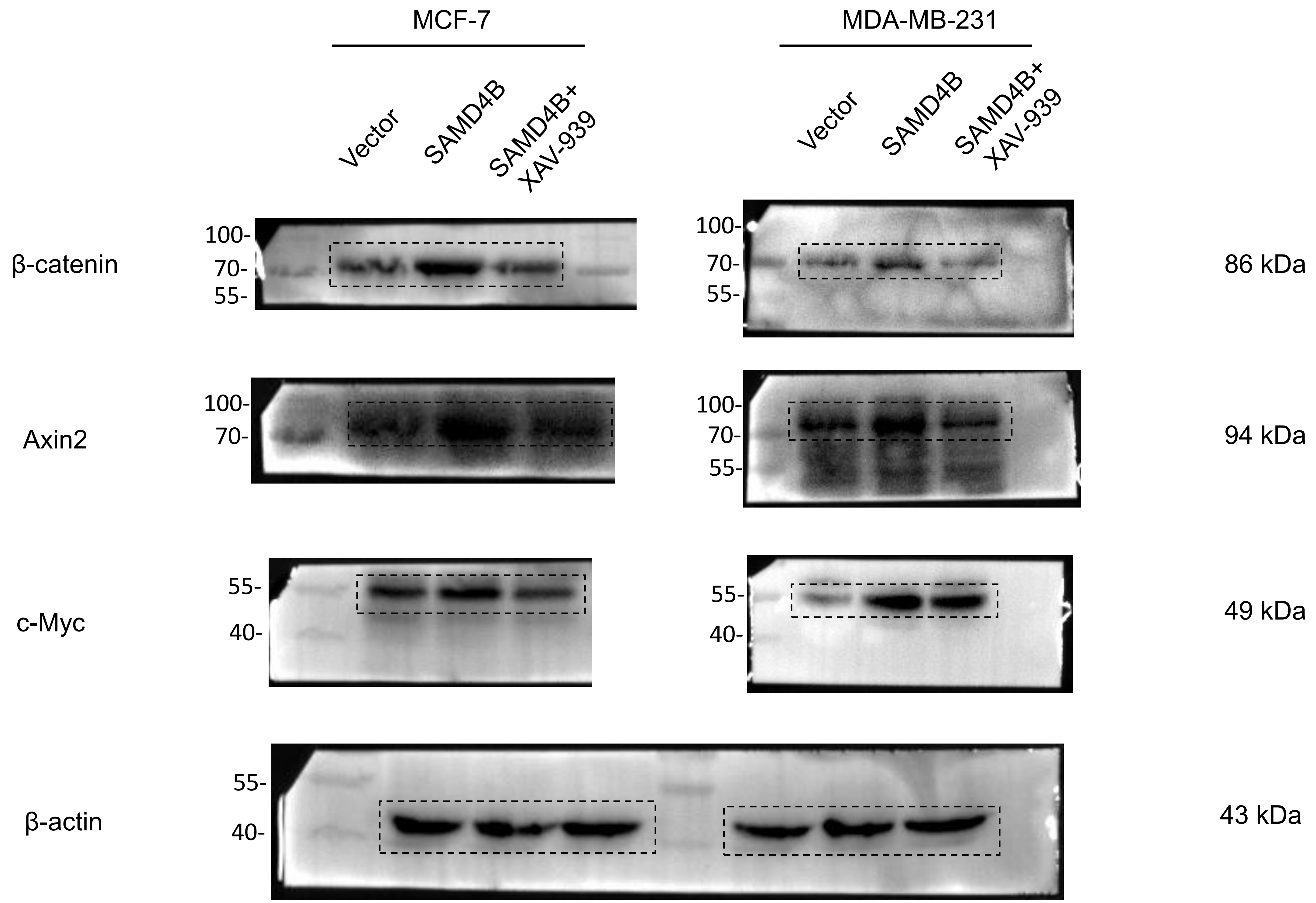

(H) Original Western Blot gels for Figure 7F

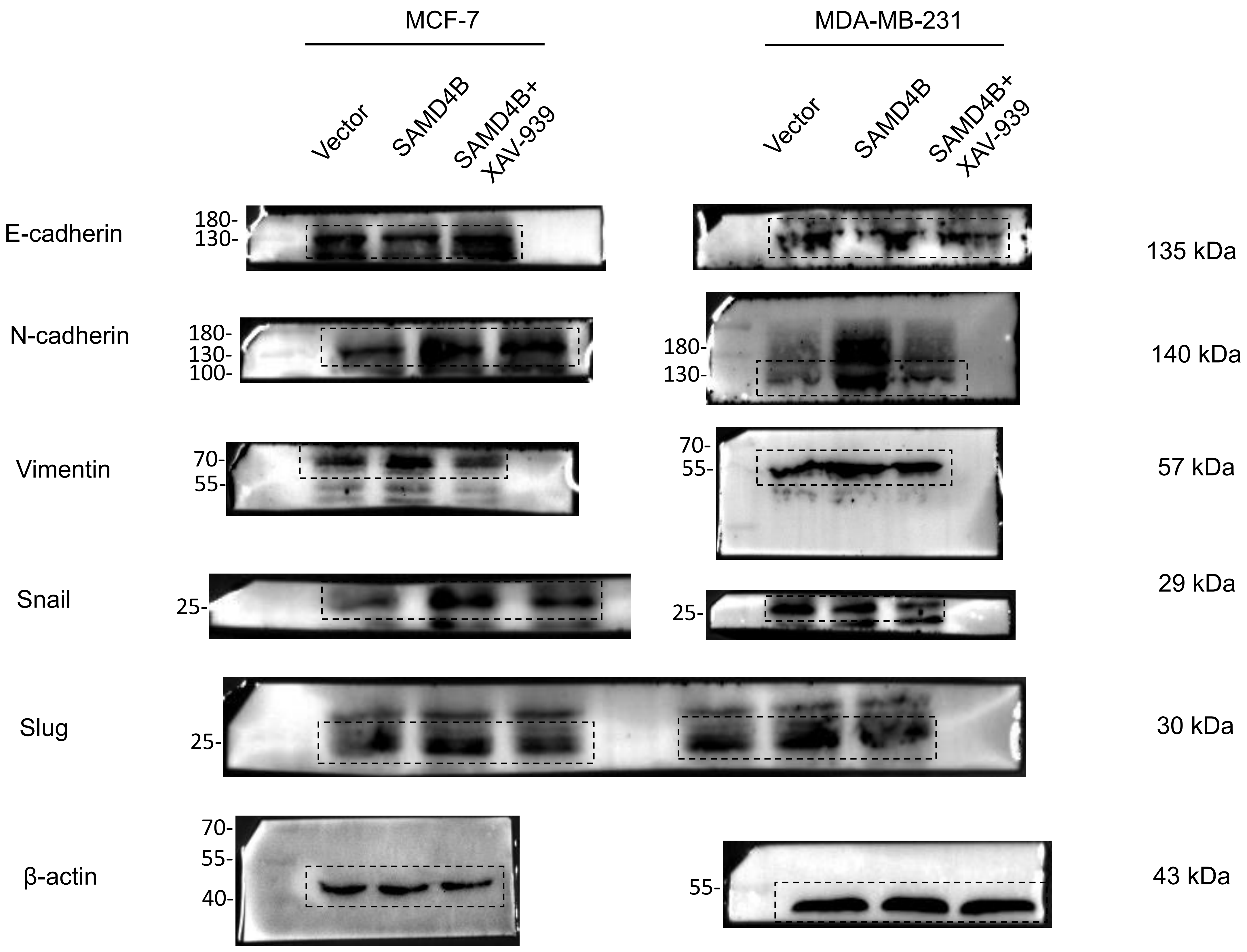

Figure S2. Original Western Blot gels corresponding to each figure. (**A**) Original Western Blot gels for Figure 1D. **(B)** Original Western Blot gels for Figure 2B and 2D. **(C)** Original Western Blot gels for Figure 3C and 3D. **(D)** Original Western Blot gels for Figure 4A,4B, 4G and 4H. **(E)** Original Western Blot gels for Figure 5G and 5H. **(F)** Original Western Blot gels for Figure 6C and 6D. **(G)** Original Western Blot gels for Figure 7B. **(H)** Original Western Blot gels for Figure 7F.
